# Artemisia Species with High Biological Values as a Potential Source of Medicinal and Cosmetic Raw Materials

**DOI:** 10.3390/molecules27196427

**Published:** 2022-09-29

**Authors:** Halina Ekiert, Marta Klimek-Szczykutowicz, Agnieszka Rzepiela, Paweł Klin, Agnieszka Szopa

**Affiliations:** 1 Chair and Department of Pharmaceutical Botany, Faculty of Pharmacy, Medical College, Jagiellonian University, Medyczna 9, 30-688 Kraków, Poland; 2 Department of Dermatology, Cosmetology and Aesthetic Surgery, The Institute of Medical Sciences, Medical College, Jan Kochanowski University, IX Wieków Kielc 19a, 25-516 Kielce, Poland; 3 Museum of Pharmacy, Medical College, Jagiellonian University, Floriańska 25, 31-019 Kraków, Poland; 4 US Army Health Clinic, Urlas Kaserne, Building 8156, 91522 Ansbach, Germany

**Keywords:** *Artemisia abrotanum*, *Artemisia absinthium*, *Artemisia annua*, *Artemisia dracunculus*, *Artemisia vulgaris*, chemical composition, pharmacological activities, cosmetic applications, safety of use

## Abstract

*Artemisia* species play a vital role in traditional and contemporary medicine. Among them, *Artemisia abrotanum*, *Artemisia absinthium*, *Artemisia annua*, *Artemisia dracunculus*, and *Artemisia vulgaris* are the most popular. The chemical composition and bioactivity of these species have been extensively studied. Studies on these species have confirmed their traditional applications and documented new pharmacological directions and their valuable and potential applications in cosmetology. *Artemisia* ssp. primarily contain sesquiterpenoid lactones, coumarins, flavonoids, and phenolic acids. Essential oils obtained from these species are of great biological importance. Extracts from *Artemisia* ssp. have been scientifically proven to exhibit, among others, hepatoprotective, neuroprotective, antidepressant, cytotoxic, and digestion-stimulating activities. In addition, their application in cosmetic products is currently the subject of several studies. Essential oils or extracts from different parts of *Artemisia* ssp. have been characterized by antibacterial, antifungal, and antioxidant activities. Products with *Artemisia* extracts, essential oils, or individual compounds can be used on skin, hair, and nails. *Artemisia* products are also used as ingredients in skincare cosmetics, such as creams, shampoos, essences, serums, masks, lotions, and tonics. This review focuses especially on elucidating the importance of the most popular/important species of the *Artemisia* genus in the cosmetic industry.

## 1. Introduction

Over the past few years, *Artemisia* species have gained huge research interest due to their chemical composition and biological activities. This increase in interest is undoubtedly due to the award of the Nobel Prize in medicine in 2015 for the discovery of artemisinin—a sesquiterpenoid lactone effective in the treatment of malaria, which is found in *Artemisia annua*. In addition to *A. annua*, *Artemisia abrotanum*, *Artemisia absinthium*, *Artemisia dracunculus*, and *Artemisia vulgaris* are also popular worldwide. Their applications are even found in historical traditional medicine. Today, their chemical composition and biological properties have been extensively studied. Of particular importance is the increase in interest in the application of these species in cosmetic products [1,2].

The habitats of different *Artemisia* ssp. differ from one another and are widely distributed. Natural habitats of these species are found in Europe, Asia, North Africa, North and South America, and Australia [1,2].

For years, plants have been used as remedies mainly in areas where they occurred naturally. Today, their ethnobotanical and ethnopharmacological indications have been proved by scientific studies. There are known species, such as *Matthiola incana* and *Daphne mucronata* as well as the plants from genus *Aronia*, *Mimosa*, *Schisandra*, and many others, that have proven therapeutic effects and are common recognized phytopharmaceuticals [3,4,5,6,7]. For centuries, *Artemisia* ssp. have been considered effective in various ailments, e.g., parasitic disease, digestive ailments, irritation, and allergic rashes [8,9,10,11,12]. Currently, *Artemisia* ssp. are also used in phytopharmacology. Contemporary pharmacological studies have been focused on confirming and explaining the mechanisms of these traditionally reported activities. Of late, *Artemisia* ssp. extracts have been scientifically proved to exhibit many biological activities. Research studies have primarily focused on *A. absinthium*, which is reported to show hepatoprotective, neuroprotective, antidepressant, cytotoxic, and digestion-stimulating activities [13,14,15,16,17,18,19]. Furthermore, antitumor activity has been documented for *A. abrotanum* and *A. dracunculus* extracts [20,21]. *A. vulgaris* and *A. dracunculus* have been shown to have an interesting biological effect on the endocrine system. *A. dracunculus* normalizes the profile of thyroid hormones, whereas *A. vulgaris* shows estrogenic activity [22,23,24]. One of the most important biological properties of *Artemisia* ssp. is their antiprotozoal activity, which has been proved for *A. absinthium*, *A. annua*, and *A. dracunculus* extracts [25,26,27,28,29,30,31,32,33,34,35,36,37].

Furthermore, the use of *Artemisia* ssp. in the production of cosmetic products has been increasing significantly. They are used as ingredients in cosmetic products for skin and hair care and also in perfumes. Extracts of *A. abrotanum* and *A. absinthium* have scientifically proven effects against acne-causing bacteria (*Propionibacterium acnes*). In addition, *A. abrotanum*, *A. absinthium*, *A. annua*, *A. dracunculus*, and *A. vulgaris* extracts have been characterized by antioxidant activity. These properties are highly important due to their possible antiaging effect in cosmetic products [20,38,39,40,41].

While compiling this review, great efforts were invested to present the qualities of the most popular *Artemisia* ssp. (*A. abrotanum*, *A. absinthium*, *A. annua*, *A. dracunculus*, and *A. vulgaris*) in detail, with a particular emphasis on their cosmetological properties. In this review, chemical composition, biological activities, traditional and contemporary medicinal uses, and the safety of the abovementioned species are discussed.

## 2. Materials and Methods

A detailed literature review that included papers published from 1978 to 2022 was carried out. Several databases, such as Scopus, Google Scholar, PubMed, were explored in order to collect information on *A.* *abrotanum*, *A. absinthium*, *A. annua*, *A. dracunculus*, and *A. vulgaris*. Various publications, chapters and books were consulted. The species names and the synonyms names were used as keywords. The scientific names and their synonyms were validated using a standard database—The World Flora Online [42].

## 3. General Characteristics of *Artemisia* Species

*Artemisia* ssp. gained huge research attention in 2015, when the Nobel Prize in medicine was awarded for the discovery of artemisinin [1,2], a sesquiterpenoid lactone isolated from *A. annua* (annual mugwort), proving its effectiveness in the treatment of malaria. Subsequently, the chemistry and biological activity of other *Artemisia* species have gained increasing attention [8,9,10,11,12]. There are more than 300 *Artemisia* species. Furthermore, some *Artemisia* ssp. have many synonymous Latin names. In this review, the five most popular *Artemisia* ssp. worldwide from a phytopharmacological point of view were studied: *A. abrotanum*, *A. absinthium*, *A. annua*, *A. dracunculus*, and *A. vulgaris*.

The natural habitats of *Artemisia* ssp. are wide-ranging. *A. abrotanum*, *A. absinthium*, *A. annua*, *A. dracunculus*, and *A. vulgaris* are found mainly in Asia and Europe. However, the distribution of these species may differ from one another. *A. abrotanum* and *A. dracunculus* grow in Central Asia and Mediterranean countries. Additionally, *A. abrotanum* grows in Central and Northwestern Europe [1,43,44,45,46,47], whereas *A. dracunculus* grows in Eastern Europe and North America [2,47]. In West Asia, the natural habitats of *A. absinthium* and *A. annua* are found. The natural habitats of *A. absinthium* and *A. annua* are found in North and South Africa and Australia. The species *A. vulgaris* is widespread, as it is found in many areas of Asia, including the Himalayas, throughout Europe, and in warm regions of North America [44,45,46,48] (Table 1).

*Artemisia* ssp. are also artificially cultivated across the world. For instance, *A. abrotanum* is cultivated in the USA, whereas *A. absinthium* is cultivated in southern Europe, the USA, and Brazil [8,9,49,50,51]. The successful cultivation of *A. annua* has been carried out in many tropical countries, such as Congo, India, and Brazil. It is also an industrial crop in China, Kenya, Tanzania, and Vietnam. The species *A. dracunculus* is widely cultivated in North and South America, Asia, and Europe [52,53,54], while *A. vulgaris* is cultivated on an industrial scale in Italy, France, Brazil, Japan, and in the mountainous regions of India and Sri Lanka [55].

The mentioned species—*A. abrotanum*, *A. absinthium*, *A. annua*, *A. dracunculus*, and *A. vulgaris*—are herbaceous plants that grow up to 1.5 m in height, except for *A. vulgaris*, which can grow up to 2.5 m. The shape of the leaves may differ between species. The flowers are yellow and can be lingual and tubular. Inflorescences may be branched panicles or raceme-like. In each species described, the fruit is achenes. Detailed information on the morphological features of leaves, flowers, and fruits is presented in Table 1.

**Table 1:** Comparison of botanical characteristics and occurrence of *Artemisia* ssp.

Species	Height	Leaves	Flowers	Fruits	Occurrence
*A. abrotanum*	0.7–1.5 m [56]	Gray-green leaves with numerous covering hairs on the upper side; the smooth underside of the leaves; in the lower part of the stem are doubly pinnate with ensiform sections; in the upper parts a singly pinnate, tripartite, also with ensiform shape [57,58]	Tiny yellow tubular flowers, gathered in spherical or ovoid-spherical hanging heads, panicle forms	Small oblong achenes [57,58]	Central Asia (Armenia, Iran, and Russia), Asia Minor (Turkey), Central and North Europe Europe (e.g., Albania and Croatia) [1,8]
*A. absinthium*	0.8–1.5 m [8,9,59]	Gray-green color, densely pubescent on both sides; basal leaves with long petioles, triangular or oval blade, bi- or tripinnatisect, the lower leaves not intensely divided, and the lanceolate top leaves [8,9,59]	Capitulum inflorescences gathered in loose panicles from the axils of the leaves; light-yellow ligulate female flowers, and tubular hermaphroditic flowers [9,59]	Small achene with brown stripes [59]	Europe, West Asia, and North Africa; introduced and acclimatized in North and South America and Australia [8,9,49,50,51]
*A. annua*	0.3–1 m [10]	Alternate arrangement [60], the tripinnatisect lower leaves from petioles, the middle leaves bipinnatisect, the upper leaves sessile with lanceolate shape [61], leaf blades can be ensiform or lanceolate, the edge of the blades serrated [8]	Flower heads in raceme-like inflorescences, small, spherical, yellow-green, only tubular flowers [8,61]	Small, long achenes [60]	Southeastern Europe, Western Asia, North and South America, Australia [8,51,60]
*A. dracunculus*	0.5–1.5 m [2,62,63]	Alternate, sessile, the lower leaves tripartite at the apex, the middle and upper leaves lanceolate, tip of the leaf sharp and the leaf blade margins entire [2,62,63]	Yellow, tubular flowers in hanging, spherical capitula forming loose panicles [2,62,63]	Small achenes [2,62,63]	Central Asia, South Europe, Eastern Europe, North America [2]
*A. vulgaris*	0.5–2.5 m [8,64]	Dense and alternate, primarily in the upper parts of the stem, the lower leaves with short petioles divided into segments and feathery shape, the middle and upper ones smaller and single or double pinnate, the dorsal side of the leaves with dark green color, the ventral side whitish and tomentose [65,66]	Small, almost bare, yellowish or brown-red flowers embedded in small baskets form heavily branched panicles with numerous lanceolate bracts at the top of the shoots, inflorescences with ligulate flowers and tubular flowers [65,66]	Small dark brown shiny achenes [66,67]	Europe, Asia, abundantly in North America [57,64,66,67]

## 4. Phytochemical Characteristics of *Artemisia* Species

The *Artemisia* species discussed here differ from each other in their chemical composition; although there are some common classes of compounds, variable chemical composition has been reported for different species.

**Figure 1:**
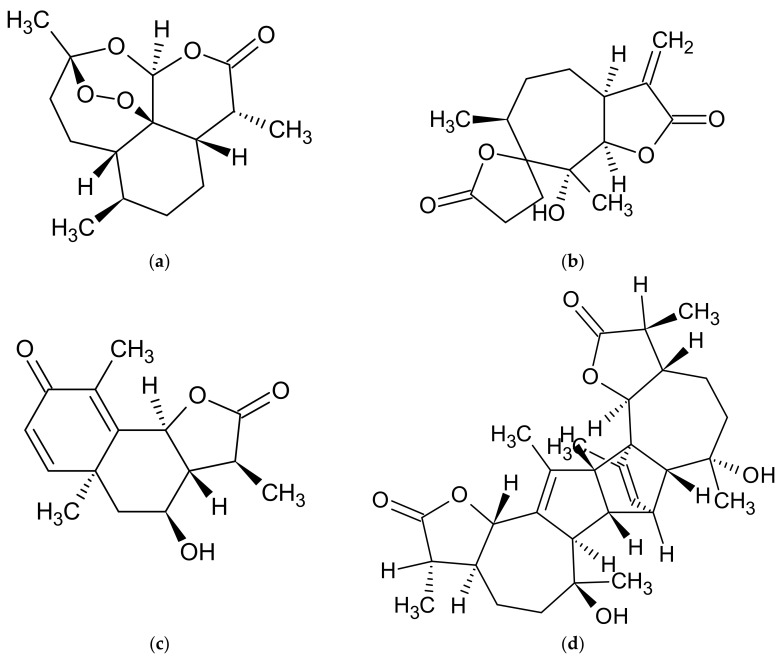
Chemical structure of sesquiterpenoid lactones found in *Artemisia* species: artemisinin (**a**); psilostachyin (**b**); artemisin (**c**); absinthin (**d**).

A common characteristic of these species is sesquiterpenoid lactones. Artemisinin (Figure 1a) is a well-known sesquiterpenoid lactone present in *A. annua*, *A. abrotanum*, and *A. vulgaris*. Artemisinin was discovered by Prof. Youyou Tu, a Chinese scientist in the field of pharmaceutical chemistry, and for this achievement and proving the effectiveness of this compound in the treatment of malaria, she was awarded the 2015 Nobel Prize in medicine [68]. In addition to artemisinin, sesquiterpenoid lactones artemisinic acid and artannuin B are found in *A. annua* [69,70,71,72,73,74], whereas in *A. vulgaris*, the presence of 1,2,3,4-diepoxy-11(13)-eudesmen-12,8-olide, psilostachyin (Figure 1b), psilostachyin C, vulgarin, and yomogin is reported. Moreover, artemisin (Figure 1c) and santonin has been identified in *A. abrotanum* [58]. Studies have reported a wide range of sesquiterpenoid lactones in the herb of *A. absinthium* [75]. The major metabolite found is absinthin (Figure 1d)—a guaianolide dimer. Other compounds, such as anabsinthin, anabsin, artabsin, and absintholide—all being absinthin isomers—are also found in high concentrations [76]. In the herb extracts of *A. dracunculus*, artemether and dihydroartemisinin have been detected [77]. Additionally, various sesquiterpenoid compounds have been reported in essential oils of the discussed *Artemisia* ssp. (Table 2) [2,11,33,54,55,57,65,73,74,78,79,80,81,82,83,84,85,86,87,88,89,90,91,92,93,94,95,96,97,98,99,100,101,102,103,104,105,106,107,108,109].

**Figure 2:**
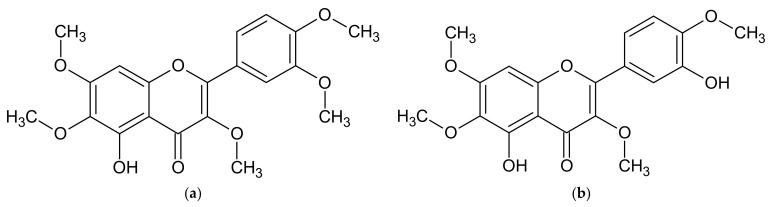
Chemical structure of flavonoids found in *Artemisia* species: artemetin (**a**); casticin (**b**).

Flavonoids are another important group of compounds found in *Artemisia* ssp. Similar to sesquiterpenoid lactones, flavonoid composition in the studied species differs from each other. The most frequently listed flavonoids characteristic of *Artemisia* ssp. are artemetin (Figure 2a) and casticin (Figure 2b), which are detected in extracts from the herb of *A. abrotanum*, *A. absinthium*, and *A. annua* [69,74,110,111]. Other *Artemisia* species also have flavonoids, such as apigenin, kaempferol, luteolin, and quercetin, as well as their derivatives, such as rutoside (Table 2).

**Figure 3:**
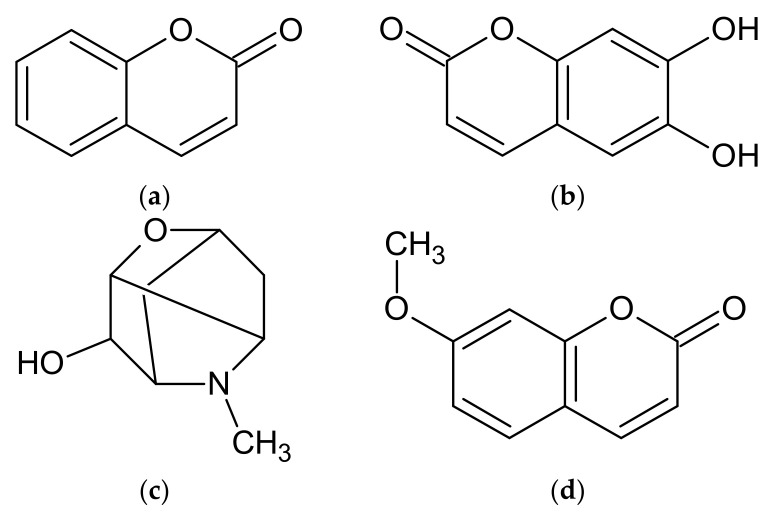
Chemical structure of coumarins found in *Artemisia* species: coumarin (**a**); esculetin (**b**); scopoline (**c**); herniarin (**d**).

Another group of metabolites found in the discussed *Artemisia* ssp. are coumarins. Several coumarins have been found in *A. dracunculus*, such as arethinol, aridiodiol, artemidiol, artemidine, artemidinol, dacumerin, and their derivatives [2,54,97,102,112,113,114,115]. In addition, the presence of coumarin (Figure 3a), esculetin (Figure 3b), scopoline (Figure 3c), and herniarin (Figure 3d) has been documented in most of the discussed *Artemisia* ssp. (Table 2) [2,54,55,84,89,97,102,111,112,113,114,115,116,117,118,119,120].

**Figure 4:**
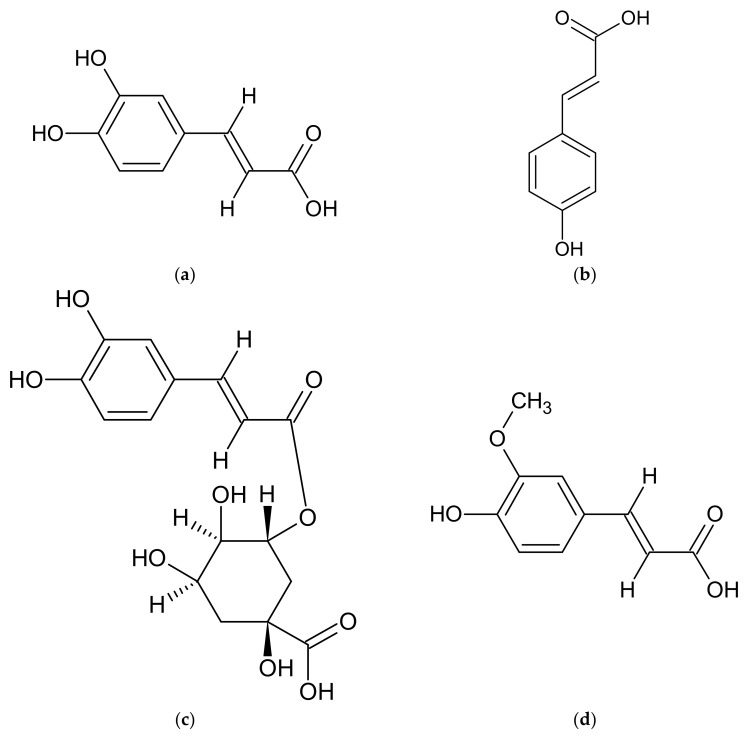
Chemical structure of phenolic acids found in *Artemisia* species: caffeic acid (**a**); *p*-coumaric acid (**b**); chlorogenic acid (**c**); ferulic acid (**d**).

Phenolic acids are another group of compounds found in *Artemisia* spp. extracts. In the most of discussed *Artemisia* ssp., the presence of caffeic acid (Figure 4a), *p*-coumaric acid (Figure 4b), chlorogenic acid (Figure 4c), ferulic acid (Figure 4d), rosmarinic acid, syringic acid, and vanillic acid has been documented [20,35,54,58,74,76,84,97,101,111,113,114,116,121,122,123,124,125,126,127,128]. In addition to the abovementioned compounds, protocatechuic acid has also been found in *A. abrotanum* and *A. vulgaris* [20,58,116,127,128], whereas gallic acid and salicylic acid have been reported in *A. absinthium* [35,76,84,121,122]. All compounds present in the discussed *Artemisia* ssp. are listed in Table 2.

**Table 2:** Chemical composition of extracts from aerial parts of *Artemisia* ssp.

Species	Sesquiterpenoid Lactones	Flavonoids	Coumarins	Phenolic Acids
*A. abrotanum*	artemisin, santonin [58]	apigenin, artemetin, casticin centaureidine, hyperoside, isoquercitrin, kaempferol, luteolin, myricetin, patuletin, rutoside, quercetin, quercetol [58]	coumarin, esculetin, herniarin, isofraxidine, scopoletin, umbelliferone [116,117]	caffeic acid, caftaric acid, *p*-coumaric acid, chlorogenic acid, ferulic acid, gentisic acid, isochlorogenic acid, protocatechuic acid, rosmarinic acid, sinapic acid, syryngic acid, vanillic acid [20,58,116]
*A. absinthium*	absintholide, absinthin, anabsin, anabsinthin, arabsin, artabin, artabsin, artenolide, caruifolin D, deacetyloglobicin, germacranolide, hydroxypelenolide, isoabsinthin, ketopelenolide, ketopepenolid-A, matricin, parishine B and C, β-santonin, santonin-related lactones [9,35,75,76,121,129,130]	apigenin, artemetin, *Artemisia* bis-isoflavonyl dirhamnoside, *Artemisia* isoflavonyl glucosyl diester, casticin, catechin, flavone, 5-hydroxy-3,3′,4′,6,7-pentamethoxyflavone, glycosides of quercetin, kaempferol, myristin, naringenin, quercetin, quercetin dihydrate, quercetin-3-rutinoside, 5,6,3, 5′-tetramethoxy 7,4′-hydroxyflavone, rutoside [9,34,35,84]	coumarin, herniarin [84,89]	caffeic acid, 5′-O-caffeoylquinic acid, chlorogenic acid, coumaric acid, *p*-coumaric acid, 1′,3′-O-dicaffeoylquinic acid, 1′,5′-O-dicaffeoylquinic acid, 3′,5′-O-dicaffeoylquinic acid, 4′,5′-O-dicaffeoylquinic acid, ferulic acid, gallic acid, rosmarinic acid, salicylic acid, syryngic acid, tannic acid, vanillic acid [35,76,84,121,122]
*A. annua*	artemisinic acid, artemisinin, artannuin B [69,70,71,72,73,74]	acacetin, apigenin, apigenin 6-C-arabinosyl-8-C-glucoside, apigenin 6-C-glucosyl-8-C-arabinoside, apigenin derivatives, artemetin, astragalin, camferol, casticin, chrysin, chrysoeriol, chrysoeriol rutinoside, chrysosplenol C, chrysosplenol D, chrysosplentin, cinaroside, cirsilineol, dihydroartemisinin, 3,5-dihydroxy-3′, 4′, 6,7-tetramethoxyflavone, 3,5-dihydroxy-6,3′, 4′-tetramethoxyflavone, 3,5-dihydroxy-6,7,4′-trimethoxyflavone, 3,5-dimethoxyquercetagentin, 3,4′-dimethyl-quercetagentin ether, ether 3-methyl-quercetin, quercetin 3-glucoside, eupatin, eupatorine, 7-O-glucoside of diosmetin, 3-O-glucoside of kaempferol, 3-O-glucoside of quercetin, 3-O-hexoside of marnsetin, isocempheride, isoquercetin, isorhamnetin, isorhamnetin derivatives, isorhamnetin 3-O-glucoside, isovitexin, jaceidin, kaempferol, kaempferol derivatives, kirsiliol, kirsimaritin, laricitrin, luteolin, luteolin derivatives, luteolin 7-O-glucoside, marnsetin glucoside, marnsetin, 8-methoxykaempferol, 3-methoxy-kaempferol glucoside, 7-methyl-luteolin ether, 3-O-methylquercetagentin, micanine, myrcetin, patulentin glucoside, quercetin, quercetin derivatives, quercetin 3-O-galactoside, quercimeritin, retina, rhamnetine, rutoside, syringetin, tamarixetine [69,74,111,119,123,124,131,132,133,134]	coumarin, esculetin, isofraxidine, *cis*-melilotoside, *trans*-melilotoside, scopoletin, scopoline, tomentin [111,118,119]	caffeic acid, 4-caffeoyl-3,5-di-succinylquinic acid, 3,5-caffeoyletherquinic acid, 3-caffeoylquinic acid, 4-caffeoylquinic acid, chlorogenic acid, coumaric acid, 3,4-di-caffeoylquinic acid, 3,5-di-caffeoylquinic acid, 3,5-di-O-caffeoylquinic acid, 4,5-di-O-caffeoylquinic acid, diferulcaffeoylquinic acid, 3,4-diferuloquinic acid, 3,5-diferuloquinic acid, 4,5-diferuloquinic acid, ferulic acid, 3-feruloquinic acid, 4-feruloquinic acid, 5-feruloquinic acid, rosmarinic acid [74,111,123,124]
*A. dracunculus*	artemether, dihydroartemisinin, [77]	anangenin, apigenin, biocovertsetin, davidigenin, 5,7-dihydroxy flavone, 2′,4′-dihydroxy-4-methoxydihydrochalcone syn, 7,3′-dimethyleriodictyol, DMC-2; 4-O-methyldavidigenin, estragoniside, estroside, 7-*O*-β*-D*-glucopyranoside, hyperoside, isoquercitrin, isorhamnetin glycosides, kaempferol, kaempferol glycosides, luteolin, luteolin glycosides, 7-methylaringenine, 7-methyleriodictiol, naringenin, patuletin hexoside, patuletin malonylrhamnosylhexoside, patuletin 3-*O*-malonylrobinobioside, patuletin rhamnosylhexoside, 5,6,7,8,4′-pentahydroxymetoflavone, pinocembrin, quercetin, quercetin glycosides, quercetin 3-*O*-rutinoside, rutoside, sacuranetine, 3,5,4-trihydroxy-7,3′-dimethoxyflavone 3,5,4′-trihydroxy-7-methoxyflavone, vicenin [2,54,97,113,114,115,125,126,135,136]	arethinol, aridiodiol, artemidiol, artemidine, artemidinol, artemidynal ether, artidin, capillarin, coumarin, dacumerin, 3,4-dehydroherniarin, (+)-(*S*,*R*)-epoxyartemidine, esculetin, esculin, herniarin, 6-demethoxycapilarisine, γ,γ-dimethylallyl ether esculetin, (+)-(*R*)-(*E*)-3′-hydroxyartemidine, 8-hydroxyartemidin, 9-hydroxyartemidine, 8-hydroxycapillarin, 4-hydroxycoumarin, isocoumarin, isovalerate capillarin, (−)-(*R*)-20-methoxydihydro-artemidine, 7,8-methylenedioxy-6-methoxycoumarin, methylenedaphnetin, 7-methyl daphnetin ether, scoparon, scopoletin, skimming [2,54,97,102,112,113,114,115]	caffeic acid, caffeoylquinic acid, chicory acid, chlorogenic acid, *p*-coumaric acid, *p*-coumaroyl-caffeoylquinic acid, *p*-coumaroyl-feruloylquinic acid, 3,5-*O*-dicaffeoylquinic acid, 4,5-di-*O*-caffeoylquinic acid, ferulic acid, ferulic acid hexoside, (*E*) 2-hydroxy-4-methoxycinnamic acid, 5-*O*-caffeoylquinic acid, hydroxybenzoic acid, 2-methoxycinnamic acid, sakuranetin, syringic acid, vanillic acid [54,97,101,113,114,125,126]
*A. vulgaris*	artemisinin, 1,2,3,4-diepoxy-11(13)-eudesmen-12,8-olide, psilostachyin, psilostachyin C, vulgarin, yomogin [55,64,137,138,139,140,141]	apigenin, chrysoeriol, diosmetin, eriodictyol, eupafolin, homoeriodictyol, hyperoside, isorhamnetin, jaceosidin, kaempferol 3-glucoside, kaempferol 7-glucoside, kaempferol 3-rhamnoside, kaempferol 3-rutinoside, luteolin, luteolin 7-glucoside, quercetin, quercetin 3-galactoside, quercetin 3-glucoside, rutoside, tricine, vitexin [23,55,142,143]	esculin, esculetin, umbelliferone [55,120]	caffeic acid, 3-O-caffeoylquinic acid, 5-O-caffeoylquinic acid, 1,5-di-O-caffeoylquinic acid, 3,5-di-O-caffeoylquinic acid, 4,5-O-di-caffeoylquinic acid, 5-feruloylquinic acid, protocatechuic acid glucoside, quinic acid [127,128]

Essential oils are the major components of the herb and leaves of *Artemisia* ssp. Studies have confirmed that the qualitative and quantitative compositions of essential oils depend on the location of the cultivation site, the salinity of the soil, and the age of the plant. The highest concentrations of essential oils are observed in two stages: at the beginning of leaf budding and at the beginning of flowering.

Monoterpenoids are abundant in the essential oils of *A. abrotanum*, *A. absinthium*, *A. annua*, and *A. vulgaris*, whereas in the essential oil of *A. dracunculus*, phenylpropanoids are predominant. The discussed species differ in terms of the composition of their essential oils. The most commonly found monoterpenoids are 1-terpineol, *trans*-piperitol, 1,8-cineole, and camphor in *A. abrotanum* [81,82,109]; thujyl alcohol esters, α-thujone, β-thujone, camphene, (Z)-epoxyocimene, trans-sabinyl acetate, and chrysantenyl acetate in *A. absinthium* [9,76]; camphene, camphor, β-pinene, borneol, and cuminal in *A. annua* [71,73,74,90,91,92,93,94,95]; sabinene, terpinen-4-ol, β-ocimene, cis-ocimene, α-trans-ocimene, limonene, α-phellandrene, β-phellandrene, (Z)-artemidin, and capillene in *A. dracunculus* [2,11,54,96,98,99,101,144,145,146]; and 1,8-cineole, sabinene, camphor, camphene, caryophyllene oxide, α-thujone, and β-thujone in *A. vulgaris* [63,65,73,88,104,105,106,107,108,147,148]. In addition to monoterpenoids, sesquiterpenoids, phenylpropanoids, and diterpenoids are found in essential oils [9,11,33,54,55,57,65,73,74,78,79,80,81,82,83,84,85,86,87,88,89,90,91,92,93,94,95,97,98,99,100,101,102,103,104,105,106,107,108,109,115,144,149,150,151]. Phenylpropanoids are detected in the essential oils of *A. abrotanum*, *A. absinthium*, and *A. dracunculus*, among which estragole, elemicine, eugenol, and their derivatives are the most common [11,54,80,82,89,97,98,99,100,101,102,103,109,115,144,149,150]. Moreover, triterpenoids and spiroterpenoids are reported in the essential oil of *A. abrotanum* [82,109], whereas triterpenoids alone are reported in *A. dracunculus* [54]. All compounds found in the essential oils of the discussed *Artemisia* species are listed in Table 3.

## 5. Applications in Medicine

### 5.1. Ethnopharmacological Uses of Artemisia Species

*Artemisia* ssp. have for long been used in the traditional European, Asian (mainly Chinese and Hindu medicine), and South American medicines (Table 4). The uses of infusions, extracts, and tinctures, as well as dried parts of plants, are here reported. In the traditional medicines of China and South America, *A. abrotanum*, *A. annua*, and *A. vulgaris* have been used, especially in malaria treatment [8,71,156].

In the European traditional medicine, *Aboratani herba* has been used in liver diseases, such as atony, the contractile states of the bile ducts, and the stagnation of or insufficient bile secretion. *Artemisia* ssp. infusions are recommended as an aid in cases of anorexia, flatulence, and hypoacidity [157]. *A. abrotanum* leaves have been used to stimulate menstruation [20].

The flowers of *A. absinthium* have been used in the European folk medicine to treat parasitic diseases and digestive ailments. The herb of this species was used to treat jaundice, constipation, obesity, splenomegaly, anemia, insomnia, bladder diseases, menstrual cramps, and injuries and nonhealing wounds [8,9,10]. The tincture of *A. absinthium* is a valuable tonic and digestive aid. Similarly, *A. absinthium* is used in the traditional Hindu medicine (Unani), in the drug “Afsanteen”, which is used to treat chronic fever, hepatitis, and edema [9].

All the parts of *A. annua* are used in the traditional medicines of China and India, such as flowers, leaves, stems, seeds, and essential oils. They are used to treat jaundice, bacterial dysentery, fever, bleeding wounds, and hemorrhoids [71,158].

In European traditional medicine, *A. dracunculus* is used to treat ailments of the digestive system and as an appetite and digestive stimulant [54,159]. According to the Hindu traditional medicine (Ayurveda), *A. dracunculus* is effective in the treatment of helminthiasis and intestinal smooth muscle spasms and in the regulation of the menstrual cycle [54,160]. In Arabic countries, *A. dracunculus* is used in the treatment of gingivitis and foot and mouth disease, whereas in Central Asia, including Russia, it is used to treat irritation, allergic rashes, and gastritis [11,12].

In European folk medicine, the oral administration of *A. vulgaris* stimulates the secretion of gastric juice. The species *A. vulgaris* is also used as a relaxant for the gastrointestinal tract and bile ducts and for relieving colic [55], whereas its laxative effect is observed in the treatment of obesity. In traditional Hindu medicine (Unani), many preparations based on *A. vulgaris* are used. These preparations are recommended for liver inflammation and obstruction, treating enlarged liver or spleen and nephrolithiasis, chronic fever, and dysmenorrhea [161]. In the Asian medicine, *A. vulgaris* is often used in the treatment of gynecological diseases [162,163]. Furthermore, *A. vulgaris* is recommended for inducing labor or miscarriage [164].

**Table 4:** Ethnopharmacological uses of *Artemisia* species.

Species	Traditional Activity	Traditional Medicine	References
*A. abrotanum*	liver diseases contractile states of the bile ducts stagnation of or insufficient bile secretion stimulate menstruation	Europe	[20,157]
*A. absinthium*	parasitic diseases and digestive ailments treating jaundice treating constipation treating obesity treating splenomegaly treating anemia treating insomnia treating bladder diseases treating menstrual cramps treating injuries and nonhealing wounds	Europe	[8,9,10]
	digestive aid treating chronic fever treating hepatitis treating edema	Hindu medicine (Unani)	[9]
*A. annua*	treating jaundice treating bacterial dysentery treating fever treating bleeding wounds treating hemorrhoids	China and India	[71,158]
*A. dracunculus*	ailments of the digestive system an appetite and digestive stimulant	Europe	[54,159]
	treatment of helminthiasis treatment intestinal smooth muscle spasms treatment in the regulation of the menstrual cycle	Hindu traditional medicine (Ayurveda)	[54,160]
	treatment of gingivitis treatment foot and mouth diseases	Arabia	[11,12]
	treating irritation treating allergic rashes treating gastritis	Central Asia	[11,12]
*A. vulgaris*	stimulates the secretion of gastric juice relaxant for the gastrointestinal tract and bile ducts relieving colic laxative effect in the treatment of obesity	Europe	[55]
	liver inflammation and obstruction, treating enlarged liver or spleen treating nephrolithiasis, treating chronic fever treating dysmenorrhea recommended for inducing labor or miscarriage	Hindu medicine (Unani)	[161,164]

### 5.2. Contemporary Phytotherapy

There are many monographs published by the European Medicines Agency (EMA) on the homeopathic preparations of *A. abrotanum* [165]. Moreover, *A. abrotanum* is included in homeopathic medicine according to the French Pharmacopoeia. These preparations are recommended for the treatment of the inflammation of the colon, rosacea, frostbite, inflammation of the lymph nodes, mucous membranes, and anxiety [166,167,168].

Among *Artemisia* ssp., *A. absinthium* herb (*Absinthii herba*) alone has the pharmacopoeial monograph in the newest tenth edition of the European Pharmacopoeia [59]. The raw material is the herb collected from young plants—in their first year of vegetation, butt-end leaves are cut off—and from older plants with sparsely leaved, flowering shoot tips. The essential oil content of this raw material is standardized; this content must not be less than 2 mL/kg in the dried herb. Moreover, the bitterness index of the raw material must not be less than 10,000 [59]. In addition, the European Pharmacopoeia and the French Pharmacopoeia have classified the fresh, flowering herb of *A. absinthium* as a homeopathic raw material. The tincture produced should contain a minimum of 0.05% (*w*/*w*) of derivatives of hydroxycinnamic acid, expressed in terms of chlorogenic acid [169]. In the homeopathic medicine, the plant is recommended for hallucinations, nightmares, nervousness, insomnia, dizziness, and epileptic seizures [170]. Additionally, *A. absinthium herba* has been discussed in a monograph in the German Pharmacopoeia. The herb of *A. absinthium* is indicated for the loss of appetite, digestive problems, and bile secretion disorders [171,172,173]. Furthermore, the German Pharmacopoeia also mentions a tincture from the herb [174]. Homeopathic preparations from the herb of *A. absinthium* have been discussed in monographs published by EMA [165]. The species *A. absinthium herba* is recommended as the raw material in the temporary loss of appetite, mild dyspepsia, and gastrointestinal disorders. It can be used in different forms, e.g., finely divided or powdered herbal substance, fresh juice, or tincture from the herb. Commercial herbal preparations are made in solid or liquid forms, and the finely divided herb is used in herbal teas. Moreover, the herb of *A. absinthium* has been discussed in a monograph of the ESCOP (European Scientific Cooperative on Phytotherapy). It can be used in digestive disorders and anorexia [175].

There are no monographs in European pharmacopeias describing *A. annua*. However, monographs of *Artemisiae annuae folium* are found in the Chinese Pharmacopoeia and the Vietnamese Pharmacopoeia [176,177]. The raw material of *Artemisiae annuae folium* is standardized for the artemisinin content, which cannot be lower than 0.7% of dry weight. It is recommended for the treatment of fever of various origins and malaria [10]. It is worth noting that *Artemisiae annuae herba* is included in the International Pharmacopoeia published by the WHO [10].

It must be noted that *A. dracunculus* is not a pharmacopoeial species, and it is used only in the traditional medicine.

The species *A. vulgaris* is classified as a homeopathic raw material in the tenth edition of the European Pharmacopoeia [178] and in the French Pharmacopoeia [179]. Its preparations are recommended for the treatment of irregular menstrual cycles and menopausal symptoms [66], and nervous disorders such as sleepwalking, seizures, epilepsy, and anxiety [170]. In addition, *A. vulgaris herba* has been discussed in a monograph in the German Pharmacopoeia. It abovementioned uses are listed only in the traditional medicine, and it has been emphasized that the effectiveness of *A. vulgaris* preparations had not been confirmed; hence, they are not recommended for therapeutic uses [172]. Furthermore, *A. vulgaris* has been described in a monograph published by the European Food Safety Authority (EFSA) [148].

*Artemisia* ssp. extracts have scientifically proven biological activities. Most of the studies are concentrated on *A. absinthium*, which have confirmed that *A. absinthium* extracts have an influence on the digestion system, due to their appetite-stimulating, antiulcer, and hepatoprotective effects, among others activities [13,19,180,181,182,183,184]. Additionally, they have also shown, inter alia, cytotoxic, anthelmintic, antiprotozoal, analgesic, immunostimulating, cytotoxic, neuroprotective, and antidepressant activities [14,15,16,17,18,25,26,30,31,32,33,34,35,36,37,86,122,130,185,186,187,188,189,190,191].

Antitumor activity was confirmed in *A. abrotanum* leaf extracts and essential oil components [20,168]. Flavonoids from *A. abrotanum* are reported to relieve the symptoms of allergic rhinitis [117]. The extract from the leaves has shown antiparasitic activity [192].

Extracts of *A. annua* essential oil and its components have scientifically proven effects, such as immunosuppressive, cytotoxic, analgesic, neuroprotective, and antimalarial properties, and have shown auxiliary effects in obesity treatment [91,93,123,131,193,194,195,196,197,198,199,200,201,202,203].

Studies have confirmed the antitumor, hepatoprotective, immunosuppressive, antidepressant, and hypoglycemic activities of *A. dracunculus* extracts and their components. [21,40,112,114,149,204,205,206,207].

Hepatoprotective, anthelmintic, cytotoxic, analgesic, hypolipemic, antihypertensive, and bronchodilatory activities have been reported for of *A. vulgaris* extracts, inter alia [138,142,186,208,209,210,211,212,213,214,215].

Scientifically proven biological activities and mechanisms of action of *Artemisia* ssp. are presented in detail in Table 5.

## 6. Cosmetic Potential of *Artemisia* Species

### 6.1. From the History of Cosmetic Uses of Artemisia Species

In the twenty-first century, the terms “cosmetics” and “cosmetology”, meaning “the art of body care”, refer to not only a wide range of products and application techniques but also a multisector industry for which modern medical laboratories work, exclusively focusing on the beautifying aspect of the manufactured preparations. For this reason, the analysis of the historical sources in terms of possible cosmetic uses must be adapted to the time when the preparation was made or described.

In the therapeutic portrait of mugwort *A. vulgaris*, three forms of external application are shown, which can now be treated also as elements of cosmetic care: sit-ups, diaphoretic baths, and leg compresses [222,223,224,225].

Diaphoretic baths are used to regulate menstrual bleeding, especially in women experiencing trouble becoming pregnant.

Leg wraps, in the form of ointments or compression dressings, have the longest history of indication and are described in all epochs. They eliminate leg fatigue, reduce exercise pain in the lower limbs, and maintain the condition of the skin in these areas.

It is worth noting that although the use of *A. vulgaris* monopreparations without any admixtures is considered sufficient for each of the above indications, some authors have also provided recipes with an extended composition, e.g., with the addition of mugwort, chamomile flowers, mint pour, or lemon balm.

Most of the sources confirming the cosmetic use of *Artemisia* spp. refer to mugwort wormwood (*A. absinthium*).

In ancient Rome, wormwood (“artemisia” in Latin) was an ingredient in hair dyes. The use of wormwood ash, mixed with rose ointment, to anoint the hair to make it black, was mentioned by Pliny the Elder in Historia Naturalis (HN 15.87) [226].

Elagabalus, the Roman emperor who reigned from 218 to 222 AD, provided information about bathing in water flavored with rose petals and wormwood in another ancient work Scriptores Historiae Augustae [226].

According to Dioscorides (first century), a Greek physician and botanist, who is the author of the work on medicinal substances “Peri hyles iatrikes” (“De materia medica”), mugwort wormwood (“Apsinthion bathypicron” in Greek) should be used with water for blemishes formed at night and mixed with honey for bruises, eye problems, and rheumy ears. Wormwood cooked in raisin wine (“passum” in Latin) helped to ease eye pain, which was applied in the form of a soothing poultice and rubbed with oil to protect against insect bites [227].

Similar descriptions of the cosmetic uses of mugwort were also reported in the so-called renaissance Polish herbaria (herbaria), which were based on the works of ancient and medieval botanists.

Szymon Syreński (Syrenius), the author of the Herbarium published in 1613, provided much information on the nurturing and healing properties of *A. absinthium* L. According to him, fresh wormwood, grated with honey and ground caraway seeds, removes dark circles below the eyes and bruises all over the body; in the case of bruises covered with blood, crushed wormwood, sprinkled with wine on a hot brick, should be used. It helps with itchy pimples, scabies, and lichens when grated with coating, cumin, and white pepper and served with white wine. A daily intake of wormwood juice mixed with wine and drunk is reported to remove skin problems (impetigo). Wormwood is also effective in eye ailments, such as redness, swelling, and pain. For bloodshot eyes, either a poultice of mashed wormwood mixed with the white of fresh egg or eye drops made of wormwood with breast milk and a little rose vodka was used. The hair care benefits of wormwood are listed in the Herbarium of Syrenius: washing with wormwood boiled in water can remove dandruff and scabs on the head and frequent washing with wormwood cooked with a tree (*A. abrotanum* L.) can treat baldness. Wormwood also repels lice, fleas, and clothing moths. Mermaid also wrote that wormwood cooked in vinegar can be used as a mouthwash to remove unpleasant odors [228].

Information on the use of *A. absinthium* in cosmetology was also found at the beginning of the nineteenth century. In 1805, a work by a pharmacist, professor of chemistry, and pharmacognosy, J.B. Trommsdorf (1770–1837), was published, entitled “Kallopistria, oder die Kunst der Toilette für die elegante Welt” (Wien, 1805), containing the first monographs on *A. absinthium* with regard to their cosmetic use. Trommsdorf mentioned wormwood (*A. absinthium*) leaves, used in perfume production, and tarragon vinegar (*A. dracunculus*) as raw materials for cosmetic products [229].

### 6.2. CosIng Database

Of late, *Artemisia* ssp. raw materials have been increasingly appearing in cosmetic products.

Information about forms of *Artemisia* available in cosmetology is provided in the European Union Special Cosmetic Ingredients database CosIng (Table 6) [230].

Two forms of *A. abrotanum* are listed in the CosIng database, which show skin conditioning, skin protecting, and moisturizing activities.

In cosmetics, six forms of *A. absinthium* are reported, and they are reported as having antimicrobial, perfuming, skin conditioning (emollient), and hair conditioning activities. Moreover, *A. absinthium* filtrate obtained after fermentation of the leaves by *Lactobacillus* spp. is used in cosmetology.

Eleven forms of *A. annua* are listed in CosIng, which show skin conditioning, fragrance, perfuming, antiseborrheic, antioxidant, and skin protecting activities. In addition, it has been reported in CosIng that *A. annua* can be used as a cosmetic ingredient in the callus culture extracts of antimicrobial, antioxidant, hair conditioning, skin protecting, and skin conditioning activities. After the fermentation of its leaves by a microorganism, e.g., *Aspergillus* spp., *Bacillus* spp., *Lactobacillus* spp., and *Leuconostoc* spp., *A. annua* herb extracts are also used as a filtrate. Essential oils possessalso the important position.

According to CosIng, *A. dracunculus* can be used in six forms, which have skin conditioning, perfuming, and fragrance properties.

In cosmetology, *A. vulgaris* can be used in nine forms as skin conditioning, perfuming, antioxidant, and skin protecting ingredients. In addition, original cosmetic ingredients, such as filtrates obtained by fermentation with bacteria (*Bacillus* spp., *Lactobacillus* spp.) or fungi (*Saccharomyces* spp.) deserve attention [230] (Table 6).

### 6.3. Potential Cosmetic Biological Activities of Artemisia ssp. Confirmed by Scientific Studies

*Artemisia* ssp. as cosmetic ingredients are subject of numerous studies (Table 7). Essential oils or extracts of *Artemisia* ssp. discussed in this review have antibacterial, antifungal, and antioxidant activities [14,20,38,39,58,84,85,87,88,91,92,93,122,168,201,212,217,232,233,234,235,236,237,238].

From a cosmetic point of view, a very interesting scientifically proven activity against *P. acnes* strains has been reported for the extracts from the herb of *A. abrotanum* and *A. absinthium*. Studies have shown that these extracts can be used to create new therapeutic and cosmetic products for the treatment of acne and for skincare [233].

It has also been demonstrated that the antioxidant activity of *Artemisia* ssp. extracts is conditioned mainly by the presence of flavonoids and other polyphenol compounds. This antioxidant activity is very important as it is related to the antiaging effect in cosmetic products [20,38,39,40,41].

Extracts of *A. absinthium*, *A. annua*, *A*. *dracunculus*, and *A. vulgaris* have also shown scientifically proven anti-inflammatory activities [86,126,191,239,240,241,242,243].

Moreover, *A. vulgaris* herb extracts have been reported to help in decreasing skin and eye sensitivity [244].

In the Philippines, *A. absinthum* and *A. vulgaris* are traditionally used to treat skin diseases and ulcerative sores. An entire plant is made into a decoction and is used as a wash for many kinds of wounds and skin ulcers. The dried leaves are cut into small fragments to help induce a more rapid healing of wounds and are used in eczema, herpes, and purulent scabies [245].

The methanolic extracts of aerial parts of *A. absinthium* have been tested for the sun protection activity. Studies have indicated that *A. absinthium* extracts have a higher value of SPF in comparison with other species, such as *Sambucus nigra*, *Sambucus ebulus*, *Orobanche orientalis*, *Vicia faba*, *Albizzia julibrissin*, *Danae racemosa*, and *Echium amoenum*. These activities are significantly correlated with the phenolic and flavonoid content, which was also studied [246].

Recent studies have investigated the efficacy and safety of a nail gel containing glycerin and *A. abrotanum* extract in the treatment of nail plate surface abnormalities. The findings of these studies have confirmed a significant reduction in roughness and an increase in smoothness. These values were observed after 2 and 8 weeks of using the preparation [247].

Studies of *A. vulgaris* extracts have focused on the antioxidant effect against the oxidative stress caused by UV radiation, which was tested on hairless mouse skin. The *A. vulgaris* extract and, for comparative purposes, a lotion as well as ascorbic acid were applied on mouse skin before exposure to UV radiation. The animals were then irradiated with increasing doses of UV-B for 4 weeks. Results suggested that the *A. vulgaris* extract was more effective than ascorbic acid extract in protecting hairless mouse skin from photoirradiation and that it can be used as a potential antiaging cosmetic ingredient [248].

**Table 7:** Cosmetic and potentially cosmetic properties of *Artemisia* species.

Direction of Activity	Species	Extract/Essential Oil	Part	Classification	Compounds	Modal/Assay	Short Description of Studies Performed	References
**Antibacterial and antifungal activity**	*A. abrotanum*	Ethanol	Aerial parts	nt *	nt	Cup plate method	Lethal effecton the bacteria *Bacillus stearothermophilus* (MIC = 250 µg/mL), *Klebsiella pneumoniae* (MIC = 250 µg/mL), *Micrococcus luteus* (MIC = 500 µg/mL), *Pseudomonas cepacian* (MIC = 500 µg/mL), and *Salmonella typhi* (MIC = 125 µg/mL), and the fungi *Candida albicans* (MIC = 250 µg/mL), *Saccharomyces cerevisiae* (MIC = 125 µg/mL), and *Trichosporon beigelii* (MIC = 125 µg/mL).	[232]
		Essential oil	Aerial parts	nt	nt	In vitro/diffusion well agar method (*Escherichia coli, Proteus vulgaris*, *Pseudomonas aeruginosa*, *Staphylococcus aureus)*/paper disc diffusion method (*Candida albicans*)/	Inhibition of the growth of *Escherichia coli* (inhibition zone diameter = 16 mm), *Proteus vulgaris* (inhibition zone diameter = 18.89 mm), *Pseudomonas aeruginosa* (inhibition zone diameter = 10.33 mm), *Staphylococcus aureus* (inhibition zone diameter = 20 mm), and *C. albicans* by components of *A. abrotanum* essential oil and essential oil. Some activity against *Aspergillus flavus* Lethal effect of the essential oil of *A. abrotanum* herb on *C. albicans* (inhibition zone diameter = 20.0 mm).	[80,168,237]
		Methanol	leaves	nt	nt	A microtiter plate-based protocol (microdilution)	Inhibition of the growth of the bacteria *Bacillus cereus* (MIC = 0.41 mg/mL), *E. coli* (MIC = 0.39 mg/mL), *Listeria monocytogenes* (MIC = 0.45 mg/mL), *Micrococcus flavus* (MIC = 0.57 mg/mL), *P. aeruginosa* (MIC = 0.47 mg/mL), and *S. aureus* (MIC = 0.38 mg/mL), and the fungi *A. flavus* (MIC = 0.39 mg/mL), *Aspergillus niger* (MIC = 0.78 mg/mL), *Aspergillus ochraceus* (MIC = 0.55 mg/mL), *C. albicans* (MIC = 0.86 mg/mL), *Penicillium funiculosum* (MIC = 0.85 mg/mL), and *Penicillium ochrochloron* (MIC = 0.86 mg/mL) by leaf extracts of *A. abrotanum.*	[20]
		Ethanol	herb	nt	nt	In vitro/micromethod of diffusion in agar	Moderate inhibition of the growth of the bacteria *Citrobacter freundii* (inhibition zones diameter = 8.81 mm), *Enterococcus faecalis* (inhibition zones diameter = 6.65 mm), *E. coli* (inhibition zones diameter = 6.44 mm), *P. aeruginosa* (inhibition zones diameter = 8.52 mm), *Streptococcus pyogenes* (inhibition zones diameter = 5.29 mm), *Streptococcus agalactiae* (inhibition zones diameter = 5.19 mm), *Streptococcus gordoni* (inhibition zones diameter = 5.89 mm); methicillin-susceptible: *S. aureus* (inhibition zones diameter = 6.34 mm)and *Staphylococcus epidermis* (inhibition zones diameter = 6.38 mm); methicillin-resistant: *S. aureus* (inhibition zones diameter = 7.20 mm) and *Staphylococcus haemolyticus* (inhibition zones diameter = 6.85 mm); and macrolides-resistant: *Propionibacterium acnes* (inhibition zones diameter = 8.71 mm) strains. Decrement of *C. albicans* (inhibition zones diameter = 5.79 mm) and *Candida tropicalis* (inhibition zones diameter = 7.09 mm) colonies and *A. niger* (inhibition zones diameter = 13.32 mm) spore germination. Synergistic action of *A. abrotanum* herb ethanolic extract with erythromycin against *S. aureus* with efflux mechanism of MLS-resistance.	[233]
	*A. absinthium*	Essential oil	Aerial parts	nt	nt	In vitro	Growth inhibition by the essential oil from *A. absinthium* and its lethal activity against *Clostridium perfringens*, *Enterobacter aerogenes*, *E. coli*, *Klebsiella oxytoca*, *K. pneumoniae*, *L. monocytogenes*, *Proteus mirabilis*, *P. aeruginosa*, *S. aureus*, and *Staphylococcus sonnei* and inhibition of growth fungi *Fusarium moniliforme*, *Fusarium oxysporum*, and *Fusarium solani.* The range of MIC values was from < 0.08 mg/mL for *P. mirabilis* and *E. aerogenes* isolated from stool and for *P. aeruginosa* and *S. aureus* isolated from wounds, up to 2.43 mg/mL for *K. oxytoca* isolated from stool.	[85,88,234]
		Ethanol	Herb	nt	nt	In vitro/micromethod of diffusion in agar	Lethal effect of *A. absinthium* extract on *B. cereus* (inhibition zones diameter = 20.40 mm), *Bacillus subtilis* (inhibition zones diameter = 14.40 mm), *Haemophilus influenzae* (inhibition zones diameter = 18.40 mm), *P. aeruginosa* (inhibition zones diameter = 7.22 mm), and *S. aureus* (inhibition zones diameter = 9.37 mm) and growth suppression in *P. acnes* (inhibition zones diameter = 7.26 mm).	[233,235]
		Essential oil	Aerial parts	nt	nt	In vitro	Growth inhibition of the bacteria *L. monocytogenes* (inhibition zone = 20 mm) and methicillin-sensitive/resistant *S. cerevisiae* var. *chevalieri* (inhibition zone = 16 mm), *S. aureus* (inhibition zone = 25 mm), and the fungi *Fusarium culmorum* (inhibition zone = 45 mm), *Fusarium graminearum* (inhibition zone = 15 mm), *F. oxysporum* (inhibition zone = 19 mm), *Rhizoctonia solani* (inhibition zone = 25 mm), and *Sclerotinia* sp. (inhibition zone = 24 mm) by *A. absinthium* essential oil.	[84,87]
			Aerial parts	Phenolic acids	Chlorogenic acid, 4,5-di-O-caffeoylquinic acid	In vitro	Some bactericidal activity of chlorogenic acid and efflux pump inhibition by 4,5-di-O-caffeoylquinic acid isolated from *A. absinthium*.	[122]
		Essential oil	Aerial parts	nt	nt	In vitro	Lethal action by essential oil *A. absinthium* against the fungi *Alternaria alternata*, *A. niger*, *Fusarium oxysporum*, *F. sambucinum*, and *F. solani* and the bacteria *Arthrobacter* spp., *Bacillus mycoides*, *Micrococcus lylae*, and *P. aeruginosa*.	[236]
	*A. annua*	Water	Leaves	nt	nt	In vitro (disk diffusion method)	Lethal activity of *A. annua* leaf extracts against *E. coli*.	[201]
		Essential oil	Aerial parts	Monoterpenoids	1,8-cineole, camphor	In vitro (disk diffusion method)	Lethal activity of essential oil and 1,8-cineol, camphor, and *Artemisia* ketone isolated from *A. annua* herb against *E. coli*, *L. monocytogenes*, *Salmonella enteritidis*, *S. typhi*, and *Yersinia enterocolitica*. Components of essential oil penetrate through the bacterial cell membrane, causing cellular dysfunction, increasing permeability of bacterial membrane and components. Low and moderate growth inhibition of the bacteria *B. cereus*, *E. coli*, *K. pneumoniae*, *Sarina lutea*, *Shigella*, *S. aureus*, and *S. enteritidis*, and fungi *Aspergillus fumigatus* and *C. albicans* by essential oil and 1,8-cineol, camphor and *Artemisia* ketone isolated from *A. annua* herb.	[91,93]
		Essential oil	Aerial parts	nt	nt	In vitro (disk diffusion method)	Essential oil inhibits growth of the bacteria *Acinetobacter baumannii*, *B. subtilis*, *E. faecalis*, *E. coli*, *K. pneumoniae*, *P. aeruginosa*, and *S. aureus*, and fungi *C. albicans*, *Candida famata*, and *C. utilis*, and also inhibits cell adhesion and reduces the expression of virulence factors.	[92]
	*A. dracunculus*	Essential oil	Herb	nt	nt	In vitro (disk diffusion method)	Inhibition of the growth of *B. cereus*, *B. subtilis*, *E. coli*, *K. pneumoniae*, *L. monocytogenes*, *M. luteus*, *P. aeruginosa*, *Salmonella* sp., *S. aureus*, *S. epidermidis*, *S. pyogenes*, *Streptococcus typhimurium*, *Shigella flexneri*, and *Shigella marcescens* under the influence of the essential oil of the *A. dracunculus* herb*. Corynebacterium diphtheriae*, *Proteus* spp., and *S. aureus* colony growth inhibition after application of the essential oil. *S. epidermidis* showing the largest zone of inhibition (21.5 mm).	[101]
		Essential oil	Leaves	nt	nt	In vitro (agar well diffusion)	Essential oil of *A. dracunculus* leaves hampers the growth of *B. cereus*, *Enterobacter cloacae*, *E. coli*, *L. monocytogenes*, *M. flavus*, *S. enteritidis*, and *S. aureus* strains. *P. aeruginosa*, *A.R P. aeruginosa*, *S. aureus*, *S. aureus MRSA* (methicillin-resistant), and *S. typhimurium* colonies growth inhibition and bactericidal effect as well as inhibition of the growth of *A. fumigatus*, *A. niger*, *A. ochraceus*, *A. versicolor*, *P. funiculosum*, *P. ochrochloron*, *Penicillium verrucosum*, *Trichoderma viride*, and fungicidal activity under the influence of hydroethanolic extract of the Tarragon. The MIC value for these bacteria and fungi was determined using the essential oil at a concentration of 0.03 and 25 mg/mL.	[125,153,249]
		Hydro-ethanol	Leaves	nt	nt	In vitro (disk diffusion method)/In vivo (mice)	Hydroethanolic extract of *A. dracunculus* leaves (at dose 200 mg/kg) significantly reduces the number of colony-forming units of *C. albicans* in the liver and kidneys of mice. Inhibition of the growth of the bacteria *B. cereus*, *B. subtilis*, *E. coli*, *P. aeruginosa*, *P. vulgaris*, *S. aureus*, and *S. pyogenes*, and fungi *A. fumigatus*, *C. albicans*, and *Penicillium expansum* under the influence of hydroethanolic herbal extract. The largest zone of growth inhibition was observed for *S. pyogenes* (18 mm), and the smallest for *P. aeruginosa* (9 mm). Inhibition of the growth of the bacteria *Corynebacterium diphtheria* (MIC 5.9 mg/mL), *Helicobacter pylori* (MIC 11.75 mg/mL), *S. aureus* (MIC 0.09 mg/mL), *S. aureus MRSA* (MIC 2.35 mg/mL), and *S. epidermis* (MIC 0.363 mg/mL), after the application of infusion of *A. dracunculus* and minimal inhibition effect in *Enterococcus hirae* MIC 23.5 mg/mL) and *K. pneumoniae* colonies (MIC 47 mg/mL).	[100,126,205]
	*A. vulgaris*	Essential oil	Aerial parts	nt	nt	In vitro/paper disc diffusion method (*Candida albicans*)	Inhibitory effect of the oil fraction on the development of *E. coli*, *K. pneumoniae*, *S. enteritidis*, *P. aeruginosa*, *S. enteritidis*, *S. aureus*, and *Streptococcus mutans.* Inhibitory effect of the oil fraction on the development of *A. niger* and *C. albicans* (inhibition zone diameter = 12.5 mm).	[41,80,88,151,250,251,252]
**Antioxidant activity**	*A. abrotanum*	Ethanol	Herb	Polyphenols	Apigenin, caffeic acid, chlorogenic acid, *p*-coumaric acid, ferulic acid, gentisic acid, hyperoside, isoquercitrin, luteolin, rutoside, sinapic acid, quercitol, quercitrin,	In vitro	Moderate antioxidant activity (IC_50_ = 284.50 µg/mL) of *A. abrotanum* ethanolic extract in the test with DPPH (2,2-diphenyl-1-picrylhydrazyl).	[58]
		Essential oil	Aerial parts	nt	nt	In vitro	Reducing potential and inhibition of lipid peroxidation (82.34%, 1000 µL) by the essential oil from the herb of *A. abrotanum*.	[237]
		Methanol	Herb	Phenolic acids	Isochlorogenic acid, rosmarinic acid, quercitrin	In vitro	Reducing the potential of methanolic extract from *A. abrotanum* herb, in particular its components, rosmarinic acid, isochlorogenic acid, and quercitrin.	[20]
	*A. absinthium*	Methanol	Herb	Flavonoids, phenolic acids	nt	In vitro	Antioxidant activity of flavonoids and phenolic compounds in *A. absinthium*. In the DPPH test, the IC_50_ value for radical scavenging activity was 612 μg/mL.	[238]
		Methanol	Herb	nt	nt	In vitro/DPPH assay, FRAP assay	Methanolic extracts from *A. absinthium* herb have a significant reduction potential (IC_50_ = 9.38 mg/mL). Herb extracts reduced iron(III) ions, the EC_50_ were lower than for the ascorbic acid control	[84]
		Essential oil	Aerial parts	nt	nt	In vitro/DPPH assay, ABTS assay	*A. absinthium* essential oil has the ability to scavenge radicals in DPPH and ABTS (2,2’-azobis(3- ethylobenzotiazolino-6-sulfonian)) tests.	[88]
		Methanol	Herb	nt	nt	In vivo (mice)	Reducing properties of *A. absinthium* extract (at dose 100 or 200 mg/kg) and the ability to capture superoxide and hydrogen peroxide anions, hydroxy and nitric oxide radicals, inhibiting oxidative stress, reducing the concentration of TBARS (thiobarbituric acid reactive substances), and increasing the concentration of superoxide and glutathione dismutases.	[217]
	*A. annua*	Methanol	Leaves	Phenolic acids, flavonoids	nt	In vitro	Methanolic extracts from *A. annua* leaves have the highest concentration of phenolic and flavonoid compounds showing a reducing effect.	[39]
		Hexane, chloroform, methanol, and water	Leaves	nt	nt	In vitro	Reducing activity of *A. annua* leaf extracts in DPPH test.	[201]
		Essential oil	Herb	Monoterpenoids	1,8-cineol, and α-pinene	In vitro	Essential oil from *A. annua* herb and its components 1,8-cineol, *Artemisia* ketone, and α-pinene shows weak reducing activity in tests with DPPH, ABTS radical tests, and hydrogen peroxide.	[93]
	*A. dracunculus*	Hydro-ethanol	Herb	Flavonoids, phenolic acids	nt	In vitro	Reducing properties of the hydroethanolic herbal extract related to the presence of phenolic compounds and flavonoids. Reduction in DPPH and ABTS in the presence of phenolic compounds.	[40,100,113,125]
	*A. vulgaris*	Hydro-ethanol	Herb	Flavonoids, phenolic acids	nt	In vitro	Proved by different methods, such as DPPH (IC_50_ value was 65.5 μg/mL), lipid peroxidation, protein glycation, xanthine oxidases, ABTS, hydroxyl, superoxide, nitric oxide, ferric reducing power activity, and inhibition of lipid peroxidation by thiobarbituric acid reactive species assays. Increasing the level of ascorbic acid and glutathione.	[41,128,243,253,254]
**Anti-inflammatory activity**	*A. absinthium*	Essential oil/Methanol	Aerial parts	nt	nt	In vivo (mice)	Reduction (41%) in inflammatory edema in mice after administration of the essential oil (at dose 4 and 8 mg/kg) or methanolic extract from *A. absinthium* (at dose 300, 500, and 1000 mg/kg).	[86,191]
		nt	Aerial parts	flavonoid	5,6,3′,5′-tetramethoxy-7,4-hydroxyflavone (p7F)	In vitro, In vivo (mice)	Inhibition of the expression of nitric oxide synthase and cyclooxygenase-2, reduction in the production of prostaglandin E2, nitric oxide, and tumor necrosis factor (TNF-α), reduction in the accumulation of reactive oxygen species by 5,6,3′,5′-tetramethoxy-7,4-hydroxyflavone isolated from *A. absinthium*.	[239]
		nt	Aerial parts	Chalcone	Cardamonin	In vitro (THP-1 (monocyte cell line of acute monocytic leukaemia) and RAW 264.7 (cell line of mouse macrophages)	Cardamonin isolated from *A. absinthium* inhibits the NFĸB (nuclear factor ĸB) pathway by the direct inhibition of DNA transcription factors, which leads to reduced NO release.	[255]
		Methanol	Herb	nt	nt	In vivo (rats)	Reduction in paw edema in rats given carrageenan and venom of *Montivipera xanthina* after the application of *A. absinthium* extract (at dose 25 and 50 mg/kg).	[241]
	*A. annua*	supercritical CO_2_	Herb	nt	nt	In vivo	Reduction in pain and stiffness in joints and improvement in mobility after using *A. annua* extract (at dose 150 mg).	[242]
		Aqueous	Leaves	Phenolic acid	Rosmarinic acid		Use of aqueous extracts from *A. annua* leaves reduces secretion of proinflammatory cytokines, IL-8 and IL-6. Rosmarinic acid is largely responsible for this effect.	[119]
	*A. dracunculus*	Ethanol, Aqueous	Herb	nt	nt	In vivo (mice)	Reduction in pain sensations and xylene-induced ear edema after the administration of the ethanolic herbal extract (at dose 50 and 100 mg/kg) to mice. Aqueous extract inhibited ROS (by 1.4%), IL-8 (by 4.0 and 4.8%), and TNF-α (by 7.8 and 5.2%). Their production imitated inflammation.	[255]
	*A. vulgaris*	Methanol	Leaves	nt	nt	In vivo (rats)	Extract (at dose 400 mg/kg) caused the normalization of serum lipid profile, an increase in paraoxonase-1 activity, and a decrease in serum malondialdehyde, nitric oxide, and TNF-α level. Proved by lipoxygenase inhibitory activity assay and “Cotton Pellet Granuloma method.”	[214,243,256]
**Antiallergenic activity**	*A. vulgaris*	Aqueous	Aerial parts	nt	nt	In vivo	Decrease in skin sensitivity and eye sensitivity.	[244]

* nt—not tested.

### 6.4. Artemisia ssp. in Cosmetology

*Artemisia* ssp. are used as ingredients in skincare cosmetics, such as creams, shampoos, essences, serums, masks, lotions, and tonics. Different cosmetic brands based on *Artemisia* spp. extracts or essential oils are available worldwide.

The species *A. abrotanum* is used in the products of Australian, German, Japanese, Polish, and US cosmetic companies, whereas *A. absinthium* is very often used in the cosmetics from South Korean, Canadian, French, Russian, and USA. Furthermore, *A. annua* is used as a cosmetic ingredient in Malaysia, Swiss, Singapore, South Korea, and US cosmetic products, while *A. dracunculus* is primarily used by UK, South Korea, and US cosmetic companies (Table 8).

The essential oil of *A. dracunculus* obtained by steam distillation is widely used as an ingredient in perfumes [2]. It is also used in aromatherapy during massages and baths and in facial masks and compresses [113,145]. The essential oil of *A. dracunculus* is also very often used by prestigious fashion brands, such as the Italian *Prada*, *Versace*, *Dolce & Gabbana*; the French *Givenchy* and *Chloé*; the American *Calvin Klein* and *Tom Ford*; and many others.

The use of *A. vulgaris* is widespread in the cosmetic industry. Various companies from Canada, France, the United Kingdom, New Zealand, Norway, Russia, Indonesia, Israel, and South Korea use the *A. vulgaris* herb extract and *A. vulgaris* essential oil in the production of different cosmetics (Table 7). An original form of *A. vulgaris*—the filtrate obtained as a result of fermentation by bacteria (*Bacillus* sp., *Lactobacillus* sp.) or fungi (*Saccharomyces* sp.)—is used in cosmetic products. During fermentation, *Bacillus* sp. produces valuable physiologically active substances, such as peptides, viscous compounds (with polysaccharide structure), antioxidants, and fibrins. A combination of *A. vulgaris* and *Bacillus* sp. has been shown to enhance the effects of fermentation and to increase the antiaging and antiwrinkle effects by inhibiting the production of matrix metalloproteinase-1 and metalloproteinase-9 enzymes (decomposed of collagen) and increasing cell regeneration and collagen synthesis [35,76,84,121,122].

**Table 8:** Examples of some cosmetics based on *Artemisia* species.

*Artemisia* ssp.	Producer	Country of Origin	Trade Name	Cosmetic Form	The Form of *Artemisia* Ssp. in the Composition of the Cosmetic (INCI)	Properties of the Cosmetic According to the Producer	References
*A. abrotanum*	Alpha Keri	Australia	Breast Lift And Firm	Cream	*A. abrotanum* extract	Firming the skin of the bust	[257]
	Dr. Hauschka	German	Sensitive care conditioner	Ampoules	*A. abrotanum* flower/leaf/stem extract	The treatment in sensitive ampoules for day and night is intended for sensitive skin prone to redness and dilated blood vessels	[258]
	Laura Mercier	Japan	Infusion De Rose Moisturizing Glow Mask	Mask	*A. abrotanum* extract	Hydrates and soothes skin	[259]
	Dermika	Poland	Neocollagen M + Phytoestrogen Anti-Wrinkle Cream	Cream	*A. abrotanum* extract	Regenerating, antiwrinkle effect	[260]
	Aveeno	USA	Fresh Essentials Daily Nourishing Moisturizer SPF 30	Cream	*A. abrotanum* extract	For daily skin hydration and protection against UV radiation	[261]
	Christophe Robin Paris	USA	Cleansing Mask With Lemon	Mask	*A. abrotanum* extract	Cleans colored and thin hair	[262]
	RéVive	USA	Intensité Complete Anti-Aging Eye Serum	Serum	*A. abrotanum* extract	Antiaging decreases the appearance of lines and wrinkles and gives skin a smoother, more youthful appearance	[263]
	USANA Celavive^®^ Skincare	USA	Hydrating + Lifting Sheet Mask	Mask	*A. abrotanum* extract	Lifts, hydrates, and rejuvenates skin’s appearance	[264]
*A. absinthium*	Cera Skin Care	Canada	Timeless Retinol Night Mask	Mask	*A. absinthium* extract	Diminishes the appearance of fine lines, wrinkles, pore size, and problematic skin imperfections	[265]
	It cosmetics	France	No. 50 Serum Collagen Veil Anti-Aging Face Primer	Serum	*A. absinthium* extract	Hydrating and antiaging activity	[266]
	Natura Siberica	Russia	Super Siberica Krasnika, Amaranth & Arginine, Care Cream	Cream	*A. absinthium* herb oil	Makes hair soft and manageable	[267]
	MAN:YO	South Korea	Zaodam Sooc Essence Toner	Toner	*A. absinthium* extract	Soothes essence toner to quickly treat damaged skin	[268]
	Mizon	South Korea	Multi-function formula all in one snail repair cream	Cream	*A. absinthium* extract	Intense regenerative, moisturizing effect; narrows pores; regenerates, firms, and helps to lighten discoloration	[269]
	Bioelements	USA	Restorative Clay	Mask	*A. absinthium* oil	Cleansing skin pores	[270]
	Kiehl’s	USA	Calendula Deep Cleansing Foaming Face Wash	Foam	*A. absinthium* extract	Deeply cleansing face, cleansing foam	[271]
	MALIN + GOETZ	USA	Resurfacing Serum	Serum	*A. absinthium* oil	Smoothens, clarifies, and brightens skin	[272]
	Neogen Dermatology	USA	Vita Lightening Serum	Serum	*A. absinthium* extract	Helps to reduce the appearance of discolorations for illuminating radiance and its potent antioxidant ingredients; moisturizes and revitalizes skin	[273]
	Pixi	USA	Rose Glow Mist	Essence	*A. absinthium* extract	Strengthens skin	[274]
*A. annua*	Commonlabs	Malaysia	Vitamin E Micro Needle Spot Cream	Cream	*A. annua* extract	Antiacne activity	[275]
	Kingnature	Swiss	Artemisia creme	Cream	*A. annua* extract	Protects and cares for the skin and has a supporting effect on skin irritations and skin problems	[276]
	Su:m37	Singapore	Losec Summa Elixir Foam Cleanser	Gel	*A. annua* extract	Purifies and comforts the skin	[277]
	Dr. Oracle	South Korea	Artemisia Ultra Calming Serum	Serum	*A. annua* extract, *A. annua* leaf extract	Skin-soothing effect to irritated or sensitive skin	[278]
	MISSHA	South Korea	Artemisia Calming Ampoule	Essence	*A. annua* extract	Controls the balance of hydration and lubrication of the skin, soothes irritation and redness, controls the balance of hydration and lubrication of the skin, and soothes irritation and redness	[279]
	Neogen Dermatology	USA	Dermalogy Green Tea Moist PHA Gauze Peeling	Peeling	*A. annua* extract	Exfoliates and moisturizes skin	[273]
	PURE’AM	USA	Authentic Barrier Cream Balm	Cream	*A. annua* extract	Nourishes, repairs, and strengthens natural skin barrier	[280]
*A. dracunculus*	ESPA	Great Britain	Age-Rebel Moisturiser	Cream	*A. dracunculus* oil	Moisturizes, nourishes, and smoothens skin	[281]
	Lush	Great Britain	Dirty Shampoo	Shampoo	*A. dracunculus* oil	Cleanses hair	[282]
	Hayejin	South Korea	Blessing Of Sprout Radiance Toner	Toner	*A. dracunculus* leaf/stem extract	Brightens skin’s complexion, balances pH level, and moisturizes the skin	[283]
	Onekind	USA	Mega Multitasker All-Day Moisturizer	Cream	*A. dracunculus* oil	Hydrating, has antioxidant activity, and defends against daily damage	[284]
*A. vulgaris*	Humphrey	Canada	Mugwort Anti Acne Serum	Serum	*A. vulgaris* extract	Treats acne, reduces inflammation on acne-prone skin, soothes and moisturizes skin	[285]
	Vgam	Canada	Pure Artik	Gel	*A. vulgaris* extract	Gently removes impurities and protects skin	[286]
	Annayake	France	Makeup Remover Gel	Gel	*A. vulgaris* extract	Cleanses face and eye and removes makeup	[287]
	Cherry Brenchez	Great Britain	Venus Reviver Serum	Serum	*A. vulgaris* oil	Moisturizes skin, reduces spots and fine lines, and protects skin from sun damage	[288]
	Monuskin	Great Britain	Rosewood Reviving Mist	Essence	*A. vulgaris* oil	Refreshes and revitalizes skin	[289]
	R10 Labs	Great Britain	Hybrid Iq Shaving Gel-Oil	Gel	*A. vulgaris* oil	Softens the hair and makes it easier to shave	[290]
	Somethinc	Indonesia	AHA 7% BHA 1% PHA 3% Weekly Peeling Solution	Peeling	*A. vulgaris* extract	Helps clean clogged pores and remove dead skin cells	[291]
	Moraz	Israel	Body Oil Skin Saver	Oil	*A. vulgaris* extract	Hydrating and reduces burns, redness, itching and dryness	[292]
	Manuka Doctor	New Zealand	Apiclear Purifying Facial Peel	Peeling	*A. vulgaris* extract	Removes dead cells and stimulates cell renewal	[293]
	Skintific	Norway	Mugwort Anti Pores & Acne Clay Mask Pore Clarifying Wash Off Pack	Mask	*A. vulgaris* extract	Helps clean clogged pores, reduces skin changes, and brightens skin	[294]
	Natura Siberica	Russia	Anti Dandruff Shampoo	Shampoo	*A. vulgaris* extract	Cleanses the hair and has antidandruff properties	[267]
	Aprilskin	South Korea	Artemisia Essence Rice Toner	Toner	*A. vulgaris* extract	Calms and hydrates skin and makes skin firm	[295]
	I’m From	South Korea	Mugwort Spot Gel	Gel	*A. vulgaris* oil	Stabilizes sebum production and soothes skin	[296]
	Manyo Factory	South Korea	Herb Green Cleansing Oil	Cleansing oil	*A. vulgaris* oil	Cleanses skin	[268]
	Dermalogica	USA	Overnight Active Clearing Gel	Gel	*A. vulgaris* oil	Removes skin cells and regulates excess sebum	[297]
	Rms Beauty	USA	“re” Evolve Radiance Locking Hydrating Primer	Primer	*A. vulgaris* oil	Keeps makeup all day long	[298]

## 7. Safety of *Artemisia* ssp. Use

*Artemisia* ssp. may have limitations in use depending on other ingredients used along with them or depending on the oral intake of other ingredients simultaneously, due to which various side effects could occur.

Studies on patients taking homeopathic remedies, herbal mixtures, or single-ingredient preparations from *A. abrotanum* extracts have reported no serious adverse effects. In a previous study, only two patients out of the 236 studied showed side effects. The intake of a preparation composed of *A. abrotanum* and *Matricaria recutita* extracts was reported to cure ailments such as stomach pain and allergy [299].

The species *A. absinthium* is rich in compounds that have toxic effects, of which α- and β-thujone deserve particular attention, with α-thujone being thought to be two to three times more harmful [300]. The EFSA listed α- and β-thujone, absinthin, and anabsinthin as potentially dangerous. However, the conclusions of the EFSA report regarding *A. absinthium* contain information that the plant can be safely used as a basic substance. Furthermore, *A. absinthium* has a known toxicological profile, and its compounds that were previously considered harmful are currently being investigated as medicinal substances [300]. Nonetheless, *A. absinthium* should not be recommended if the patient has gastric or duodenal ulcers, biliary obstruction, or liver disease or if he/she is allergic to plants of the family *Asteraceae*. It should not be used during pregnancy and breastfeeding [171,175]. Studies confirmed no skin irritation after the application of undiluted *A. absinthium* essential oil [301]. The dangers of drinking absinthe are worth mentioning. Absinthe consumption initially causes the feeling of well-being and hallucinations, slowly leading to a depressive stage. In recent years, it has been speculated that absinthe causes misdiagnosed alcoholism. The symptoms characteristic of absinthism can be attributed to ethanol itself [302]. The FDA (US Food and Drug Administration) has listed *A. absinthium* as an allergenic species. The source of allergens is the pollen, which can also be present in the extracts of the plant [303].

The species *A. annua* can cause inflammation of the skin, and due to its highly allergenic pollen, susceptible people may develop allergies. Adverse effects after consumption of preparations with *A. annua* extracts are as follows: abdominal pain, bradycardia, diarrhea, nausea, vomiting, decreased appetite, flu-like symptoms, reticulocytopenia, and fever. The use of *A. annua* products is contraindicated in patients with ulcers and gastrointestinal disorders [8,304,305]. The EFSA listed *A. annua* leaves as a raw material that is not health-neutral due to the high concentration of camphor (2.58–37.5%) in the essential oil [306].

The FDA has listed *A. dracunculus* and the essential oils and extracts derived from this species as safe for use [307]. However, there have also been reports of the potential toxicity of the main components of the essential oil of *A. dracunculus* —estragole and methyl eugenol [54]. In animal studies, these components showed the adverse effects of causing, inter alia, liver tumors and neuroendocrine tumors in the glandular stomach, kidneys, and mammary glands [308]. After analyzing the available data, the EFSA has classified estragole and methyl eugenol as genotoxic and carcinogenic compounds. However, a safe threshold for the consumption of estragole and methyl eugenol has not yet been established. The EFSA recommends limiting the use of both compounds [308].

Herbal extracts of *A. vulgaris* used in therapeutic doses may not have any side effects. However, *A. vulgaris* can cause allergies, as confirmed by the FDA. Its pollen contains allergenic glycoproteins that cause type I (immediate) allergic reactions. In addition, in a few individuals, anaphylactic shock has been observed after swallowing the pollen [55,303]. The species *A. vulgaris* is also considered to be the primary cause of hay fever and allergic asthma in Northern Europe, North America, and a few regions of Asia [148,309]. People allergic to herbal ingredients from other plants of the *Asteraceae* family should avoid contact with these preparations. It has been reported that *A. vulgaris* cross-reacts with pollen from other plants as well as with food substances, such as birch, cabbage, grasses, hazelnuts, honey, pollen of the European olive, and sweet pepper, as well as with royal jelly, sunflower, kiwi, peach, mango, apple, celery, and carrot [148,310]. Apart from respiratory system ailments, allergic skin lesions have also been observed and allergic skin reactions, such as dermatitis and urticaria, may also occur [309,311,312,313].

The EFSA classified the essential oil components of *A. vulgaris*, such as α-thujone, β-thujone, camphor, and 1,8-cineol, as having potentially adverse effects on human health when taken with food or dietary supplements [306]. Therefore, *A. vulgaris* should be used with caution in patients with diabetes as it can increase blood glucose levels [148].

## 8. Conclusions

The multidirectional ethnopharmacological indications and recent popularity of artemisinin resulted in a huge increase in interest in the chemism of *Artemisia* species and in the biological activity of extracts obtained from these plants and essential oils. Research studies have confirmed their many valuable directions of biological activity, such as hepatoprotective, neuroprotective, and antidepressant effects. Some of the proven biological properties, e.g., antibacterial, antifungal, and antioxidant activities, are of particularly utility from the perspective of the cosmetic industry. In the data presented by the European Commission, in the CosIng database, the number of cosmetic raw materials approved for the production of cosmetics includes as many as 37 raw materials based on the five species characterized in this review. Cosmetics based on these raw materials are becoming more popular not only in European but also in North American and East Asian countries. 

## Figures and Tables

**Table 3:** Chemical composition of essential oil from *Artemisia* species.

Species	Sesquiterpenoids	Monoterpenoids	Diterpenoids	Triterpenoids	Phenylpropanoid Derivatives	Other Compounds
*A. abrotanum*	δ-amorphene, aromadendrene, artedouglasia C, artedouglasia oxide A, artedouglasia oxide B, artedouglasia oxide D, bicyclogermacrene, *trans*-α-bisabolen, α-bisabolol, β-bourbonene, δ-cadinene, cadinol, α-cadinol, 3-carene, caryophyllene, β-caryophyllene, caryophyllene oxide, α-copaene, davanone, davanone B cedrene, citronellol, β-copaene, α-cubebene, (*E*)-β-damascenone, davana ether, davanon ether, davanone B, *cis*-davanone, α-dehydro-ar-himachalene, γ-dehydro-ar-himachalene, β-elemene, δ-elemen, α-epi-7-epi-5-eudesmol, epi-longipinanol, 7-epi-silphiperfol-5-ene, eudesma-5-en-11-ol, α-eudesmol, β-eudesmol, γ-eudesmol acetate, farnesyl butanoate, germacrene D, germacren-D-4-ol, guaiol, α-humulene, humulene epoxide I, isospathulenol, T-muurolol, nerolidol, (*E*)-nerolidol, nordavanone, β-selinene, silphiperfol-4,7 (14)-diene, silphiperfol-5-ene, silphiperfol-5-en-3-ol A, silphiperfol-5-en-3-one A, silphiperfol-5-en-3-one B, silphiperfol-6-α-ol, silphiperfolen isomer, spathulenol [78,79,80,81,82,83,109]	borneol, bornyl acetate, camphene, camphor, 3 (10) -carene-2-ol, *trans*-carveol, *cis*-carvone, *cis*-carvyl acetate, *trans*-carvyl acetate, cembrene, *cis*-chrysanthenol, chrysanthenone, *cis*-chrysanthenyl acetate, *trans*-chrysanthenyl acetate, 1,4-cineole, 1,8-cineole, cuminyl acetate, *p*-cymenene, eugenol, geranyl isobutanoate, 2-hydroxy-1,8-cineole, isobornyl formate, isobornyl propionate, lavandulol, lavandulyl butanoate, lavandulyl caproate, lavandulyl isovalerate, limonene, ment-1,5-dien-7-ol, *p*-menth-1-en-8-ol, *p*-menth-2-en-1-ol, myrcene, linalool, β-myrcene, myrtanal, myrtenal, myrtenol, *E*-myrtenol, neryl isobutanoate, neryl propionate, β-ocimene, *E*-β-ocimene, *Z*-β-ocimene, *trans*-ocimene, *trans*-β-ocimene, *trans*-ocimenol, α-pinene, *trans*-pinocamphone, *cis*-piperitol, *trans*-piperitol, piperitone, α-phellandrene, β-phellandrene, β-pinene, 2 (10) -pinen-2-one, pinocarvone, terpenyl acetate, α-terpenyl acetate, α-terpinene, γ-terpinene, α-terpineol, 1-terpineol, 4-terpineol, *cis*-β-terpineol, δ-terpineol acetate, terpinolene, α-terpinolene, α-terpinyl acetate, 3-thujanol, α-thujenal, α-thujene, α-thujone, tricyclene, 4-tujanol, sabinaketone, sabinene, *cis*-sabinene hydrate, *trans*-sabinene hydrate, *trans*-sabinol[79,80,81,82,83,109]	lupeol, phytol isomer [80,81]	agarospirol [82]	estragol (methyl chavicol), elemicine [80,82,109]	Spiroterpenoids: methyleugenol [82,109] Jasmonates: methyl *cis*-jasmonate [79] Other: *cis*-arbusculone, *trans*-arbusculone, 1,4-dimethyl-4-propyl-2-one-1-(2)–cyclo-hexene, heptanal, hexanal, (*E*)-2-hexenal, (*Z*)-3-hexenol, α-(*E*)-ionone, isobutanoate ester of anisic acid, isopergol, *cis*-jasmone, (*Z*)-jasmone, lavender lactone, methyl *p*-anisate, 4-methylpent-2-enolide, nonanal, 1-octen-3-ol, 2-phenylacetaldehyde, 2,2,3-trimethyl-3-cyclopentene-1-acetaldehyde [80,81,82,83,109]
*A. absinthium*	*allo*-aromadendrene, ar-curcumene, α-(*E*)-bergamotene, bicyclogermacrene, α-bisabolene, (*Z*)-α-bisabolene, β-bisabolene, α-bisabolol, bisabolol oxide, bisabolol oxide B, β-bourbonene, cadinene, γ-cadinene, δ-cadinene, α-calacorene, caryophyllene, β-caryophyllene, (*E*)-caryophyllene, caryophyllene oxide, α-cedrene, α-copaene, γ-curcumene, cyperene, diepi-α-cedrene, curcumene, β-elemene, elemol, epi-β-santalene, β-eudesmol, (*E*,*E*)-farnesal, (*Z*,*E*)-α-farnesene, (*E*,*E*)-farnesyl acetate, (*E*,*E*)-farnesyl 3-methylbutanoate, (*E*)-β-farnesene, germacrene D, guaiazulene, α-gurjunene, β-gurjunene, γ-gurjunene, guaiazulene, hexahydrofarnesyl acetone, α-himachalene, α-humulene, γ-humulene, humulene oxide II, α-isocomene, β-isocomene, γ-muurolene, nerolidol, (*E*)-nerolidol, (*E*)-nerolidyl propanoate, petasitene, pethybrene, presilphiperfol-7-ene, α-santalene, β-santalene, β-selinene, silfinen-1-en, silphiperfol-6-ene, 7-α-silphiperfol-5-ene, spathulenol [33,57,84,85,86,87,88,89]	*allo*-ocimene, *Artemisia* ketone, borneol, bornyl acetate, bornyl 3-methylbutanoate, camphene, camphor, carvacrol, (*Z*)-carveol, carvone, chrysanthenol, (*Z*)-chrysanthenol, chrysanthenyl acetate, (*Z*)-chrysanthenyl acetate, 1,8-cineole, *p*-cymene, *p*-cymen-8-ol, (*E*)-epoxyocimene, (*Z*)-epoxyocimene, (*Z*)-β-epoxyocimene, (*E*)-6,7-epoxyocimene, (*Z*)-6,7-epoxyocimene, epoxyocymene, eugenol, α-fenchene, fenchone, geranial, geraniol, geranyl acetate, geranyl isovalerate, geranyl 2-methylbutanoate, geranyl 3-methylbutanoate, geranyl pentanoate, isobornyl acetate, isobornyl propanoate, iso-3-thujanol, isothujyl acetate, lavandulol, lavandulyl acetate, limonene, linalool, β-linalool, (*E*)-linalool oxide, (*Z*)-linalool oxide, linalyl acetate, linalyl butanoate, linalyl 3-methylbutanoate, linalyl propionate, lyratyl acetate, *p*-menth-3-en-9-ol, 3-methylbutanoate, myrcene, β-myrcene, neral, nerol, (*Z*)-nerolidol, neryl acetate, neryl 2-methylbutanoate, neryl 3-methylbutanoate, neryl 2-methylpropanoate, (*E*)-β-ocimene, (*Z*)-β-ocimene, phellandrene, α-phellandrene, β-phellandrene, phellandrene epoxide, pinene, α-pinene, β-pinene, 2-β-pinene, pulegone, sabinene, (*E*)-sabinene hydrate, (*Z*)-sabinene hydrate, (*E*)-sabinol, sabinyl acetate, (*E*)-sabinyl acetate, santolinatriene, α-terpinene, γ-terpinene, α-terpineol, terpinene-4-ol, terpinolene, α-terpinylacetate, α-thujene, thujol, α-thujone, β-thujone, (*E*)-thujone, (*Z*)-thujone, thujyl acetate, thujyl alcohol, thymol, tricyclene, (*E*)-verbenol, (*Z*)-verbenol [9,18,35,76,84,85,87,88,89,121]	1-(*E*)-8-isopropyl-1,5-dimethyl-nona-4,8-dienyl-4-methyl-2,3-dioxa-bicyclo(2, 2, 2)oct-5-ene, iso-1-(*E*)-8-isopropyl-1,5-dimethyl-nona-4,8-dienyl-4-methyl-2,3-dioxa-bicyclo(2,2,2)oct-5-ene, vulgarol A, vulgarol B [9,73,80]	nd ^1^	estragole, methyleugenol [89]	nd
*A. annua*	aristolon, bicyclogermacrene, β-bourbonene, β-cadinene, γ-cadinene, δ-cadinene, *cis*-cadin-4-en-7-ol, epi-α-cadinol, caryophyllene, β-caryophyllene, *cis*-β-caryophyllene, *trans*-β-caryophyllene, caryophyllene oxide, β-chamigrene, α-copaene, cubebin, β-cubeben, cubenol, β-elemen, γ-elemen, α-farnesan, *trans*-β-farnesane, germacren A, germacren B, germacren D, β-gurjunene, γ-gurjunen, humulene, α-humulene, isoledene, (–)- isolongifolen-9-one, kopaene, *trans*-β-kopaene, α-longipinene, γ-muurolene, nerolidol, nootkaton, β-selinene, selin-11-en-ol isomer, selin-3,11-dien-6α-ol, spathulenol [73,74,90,91,92,93,94,95]	*Artemisia* trien, artemisinin alcohol, artemisinin ketone, borneol, bornyl acetate, camphene, camphor, α-campholenal, *cis*-carveol, *trans*-carveol, carvone, *cis*-chrysanthenol, 1,8-cineole, cuminal, *cis*-β-O-cymene, *trans*-β-O-cymene, p-cymene, dehydro-1,8-cineol, dehydrosabinaketone, dehydrosabinene, eugenol, α-felandrene, ipsdienol, limonene, linalool, p-mentha-2,4 (8)-diene, myrcene, myrcenol, myrtenal, myrtenol, myrtenyl acetate, neryl acetate, α-pinene, β-pinene, β-pinene oxide, *trans*-pinocarveol, *cis*-pinocarveol acetate, pinocarvone, piperitone, sabinene, *cis*-sabinene hydrate, *trans*-sabinene hydrate, santolin alcohol, santolinatriene, α-terpineol, 4-terpineol, δ-terpineol, γ-terpinene, terpinolene, α-terpinolene, α-terpinene, thujen, α-thujone, α-thujene, verbenol, verbenone, yomogi alcohol [71,73,74,90,91,92,93,94,95,152]	vulgarone [90]	nd	nd	arteannuic acid, 2-H-1-benzopiranzone, benzyl benzoate, benzyl 3-methylbutanacetate, 1-dodekene, ethyl 2-methylbutanoate, eudesm-7(11)-en-4-ol, hexanal, 2-hexenyl 2-methylbutanoate, *cis*-2-hexenyl 3-methylbutanoate, isovalerate hexanoate, *cis*-jasmon, 2-methyl-2-butenyl 3-methylbutanoate, 3-methyl-3-butenyl 3-methylbutanoate nonanal, nonadecane, propyl 2-methylbutanoate [91,92,93,94,95]
*A. dracunculus*	acoradiene, ar-curcumen, α-bergamotene, bicyclermacren, α-bisabolol, β-bisabolen, *δ*-cadinene, α*-epi*-cadinol, caryophyllene, β-caryophyllene, *E*-caryophyllene, *E-*β-caryophyllene, caryophyllene oxide, α-cedrene, α-copaene, elemene, *δ*-elemene, γ-elemene farnesane, *cis*-*trans*-α-farnesene, (*E*)-β-farnesene, (*E*,*E*)-farnesane, *E*,*E-*α-farnesane, germacrene D, germacrene-D-4-ol, gleenol, α-himachalene, α-humulene, β-sesquiphellandrene, spathunelol, spatulenol, α-zingiberene [2,11,54,96,97,98,99,100,101,102,103]	allocimene, artemisinic ketone, borneol, bornyl acetate, camphene, camphor, 4-carene, ∆3-carene, carvacrol, *trans*-carveol, carvone, *E*-carvone oxide, 2-*allo*-cimene, 1,8-cineole, citronellol, citronellol acetate, citronellol formate, *o*-cymene, *p*-cymene, (*E*)-β-O-cymene *p*-mentha-1,3,8-triene, ethyl geranyl, geraniol, geranyl acetate, β-elemene, endo-isofenchene, α-fenchene, geranial, (*E*)-β-ionone, isobornyl acetate, isoterpinolene, limonene, *D*-limonene, linalool, myrcene, β-myrcene, myrtenal, nerol, neryl acetate, α*-trans*-ocimene, *allo*-ocimene, *cis-*β-ocimene, *cis allo*-ocimene, *trans* β-ocimene, *trans-allo*-ocimene, β-ocimene, β-ocimene Y, *E-*β-ocymene, *Z-*β-ocymene, *neo-allo*-ocymene *cis allo*-ocymen hydrate, phellandrene, α-phellandrene, β-phellandrene, α-pinene, β-pinene, 2-β-pinene, *p*-pinene, pinocarveol, pseudolimonene, sabinene, *trans*-sabinene acetate, *cis*-sabinene hydrate, β-sesquifelandrene, α-terpenyl acetate, terpineol, 4-terpineol, α-terpineol, α-terpinene, *γ*-terpinene, terpinolene, α-terpinolene, *trans*-4 thujanol, α-thujene, thymol, tricyclen [2,11,54,96,97,98,99,100,102,103,153]	phytol [99]	squalene [54]	(*Z*)-anethole, asarone, carpaci, dillapiole, elemycin, estragole (methylchavicol, *p*-allylanisole), eugenol, isoelemycin, isoeugenol methyl ether, isoeugenol methyl *trans*-anethole, 3-(*p*-methoxyphenyl)-1,2-propanediol, methyl eugenol, prestragol [11,54,80,97,98,99,100,101,102,103,115,144,149,150]	Isocoumarins: 3-(1*-Z*-butenyl) isocoumarin = (*Z*)-artemidin, 2-(1-*E*-butenyl)-isocoumarin = (*E*)-artemidin [2,11] Polyacetylenes: capillene, 1-phenyl-2,4-hexadiene, 1-phenyl-2,4-hexadiene-1-one [2,54,146,154,155] Other: acenaphthene, *p*-allyphenol, apiole, cinnamic acid, cinnamyl acetate, cyclohexylmorpholine, dehydro-1,8-cineole, 3-methoxycinnamaldehyde, methyl ester, methyl salicylate, myristicin, nonadecane, 1,3-oktadiene, 1-pentadecene, 5-phenyl-1,3-pentadiyne [11,102,103,146,153]
*A. vulgaris*	aromadendrene, α-*trans*-bergamotene, bicyclogermacrene, β-bisabolene, α-bisabololene, β-burbonen, α-cadinol, α-calacorene, caryophylla-4(14),8(15)-diene-5-α-ol, caryophyllene, *trans*-caryophyllene, caryophyllene oxide, α-cedrene, β-chamigrene, α-copaen, cubebene, davanone, α-elemene, β-elemene, β-eudesmol, farnesene, farnesyl acetate, germacrene D, germacrene D-4-ol, α-humulene, humulene epoxide II, humulene oxide, α-isocomene, lanceol acetate, ledol, β-longipinene, modhephene, epi-α-muurolol, (*E*)-nerolidol, petasitene, presilphiperfol-7-ene, *trans*-salvene, salvial-4(14)-en-1-one, epi-β-santalene, silphin-1-ene, 7-α-silphiperfol-5-ene, silphiperfol-5-en-3-ol (*Z*)-β-farnesene, silphiperfol-4,7(14)-diene, spathulenol, valeranone [55,65,80,88,104,105,106,107,108]	*Artemisia* alcohol, *Artemisia* ketone, artemisyl acetate, borneol, bornyl acetate, camphene, camphor, *trans*-carveol, carvone, *cis*-chrysanthenol, chrysanthenyl acetate, 1,8-cineol, cuminol, cymene, p-cymene-8-ol, dehydrosabinaketone, α-fenchen, isoborneol, isobornyl acetate, iso-3-thujanol, limonene, linalool, menthol, methyleugenol, *p*-mentha-1,4-dien-7-ol, β-myrcene, (*E*)-β-ocymen, (*Z*)-β-ocymen, α-pinene, β-pinene, *trans*-pinocarveol, piperitone, sabinaketone, sabinene, *cis*-sabinene hydrate, santolina triene, α-terpinene, γ-terpinene, α-terpineol, 4-terpineol, terpinolene, 3-thujanol, α-thujene, α-thujone, β-thujone, *cis*-thujone, thymol, *trans*-verbenol, verbenyl acetate [63,65,73,88,104,105,106,107,108,147,148]	phytol, γ-terpineol [106,108,151]	nd	nd	nd

^1^ nd—no data.

**Table 5:** Biological activities of *Artemisia* species.

Direction of Activity	Species	Extract/Essential Oil	Part	Classification	Compounds	Model/Assay	Short Description of Performed Studies	References
**Antitumor activity**	*A. abrotanum*	Essential oil	Aerial part	Monoterpenoids	Borneol, cymene, camphor, terpineol, 1,8-cineole, and aromadendrene	In vitro	Decrease in the survival of neoplastic cells of the RD (rhabdomyosarcoma). The viability of RD cells after the application of the essential oil at concentrations of 25, 50, and 100 μg/mL was 29.679%, 20.833%, and 20.256%, respectively.	[168]
		Methanolic extract	Leaves	Phenolic acids	Chlorogenic and isochlorogenic acids	In vitro	Methanolic extract of *A. abrotanum* leaves in serial concentrations of 50, 100, 200, 300, and 400 µg/mL and its components (including chlorogenic acid and isochlorogenic acid) inhibits the proliferation of cells of the Jurkat line (T-lymphoblastic leukemia line, IC_50_ = 82.64 µg/mL), MCF-7 line (breast adenocarcinoma line, IC_50_ = 71.04 µg/mL), HeLa line (cervical adenocarcinoma line, IC_50_ = 49.97 µg/mL), and HT-29 line (colorectal adenocarcinoma line, IC_50_ = 54.75 µg/mL).	[20]
	*A. dracunculus*	hexane, ethyl acetate, acetone, ethanol, acetonitrile and supercritical carbon dioxide (scCO_2_)	Leaves	Polyphenols, alkamides	nt *	In vitro (mouse lymphoma L5178YD cells)	Inhibition of the proliferation of mouse lymphoma cells (L5178YD) due to the presence of polyphenols and alkamides in leaf extracts. In the control group the tumor cell count was 17.969 × 10^6^, the acetonitrile extract from *A. dracunculus* leaves reduced the cell count to 0.1 × 10^6^.	[21]
**Alleviating allergy symptoms**	*A. abrotanum*	Essential oil and isolated flavonoids	Aerial part	Monoterpenoids, flavonoids	1,8-Cineole, davanone, linalool, centaureidine dimethylether, casticin and quercetin	In vivo	Relief of symptoms of allergic rhinitis with possible concomitant allergic conjunctivitis, symptoms of bronchial obstruction, and symptoms of exercise-induced asthma by using a nasal spray with a mixture of essential oils and flavonoids present in *A. abrotanum*.	[117]
**Digestion-stimulating activity**	*A. absinthium*	Ethanol	Herb	nt	nt	In vivo	Change in postprandial hemodynamics in the gastric digestive phase with increased hyperemia, probably due to the effects of bitter compounds contained in the herb of the plant.	[19]
**Appetite-stimulating activity**	*A. absinthium*	nt	Aerial part	nt	nt	In vivo	Enrichment of sheep fodder with silage containing *A. absinthium* increases the amount of fodder consumed, improves digestion, induces nitrogen retention, and has a positive effect on the development of microorganisms involved in nitrogen assimilation.	[180]
		nt	Aerial part	nt	nt	In vivo	Improvement in nutrient supply and digestion, faster growth, improvement in carcass quality, and the amount of fatty acids among Hanwoo steers.	[181]
**Antiulcer activity**	*A. absinthium*	carbon tetrachloride, chloroform, methanol, ethanol, hexane	Aerial part and root	nt	nt	In vivo (rats)	Decrease in gastric juice volume, reduction in gastric acid and pepsin secretion, and decrease in the digestion rate.	[182]
**Hepatoprotective activity**	*A. absinthium*	Hydro-methanol	Herb	nt	nt	In vivo (rats)	*A. absinthium* extracts (in dose 500 mg/kg) inhibit liver microsomal enzymes (20%) that are responsible for the metabolism of xenobiotics.	[183]
		Methanol	Herb	nt	nt	In vivo (rats)	Methanolic extracts from the herb of the plant (in dose 50 mg/kg) protect liver cells by reducing ALAT (alanine aminotransferase) and ASPAT (aspartate aminotransferase) levels and by reducing oxidative damage.	[13]
		Aqueous	Herb	nt	nt	In vivo (mice)	Protection of the liver due to the immunomodulatory and/or antioxidant properties of *A. absinthium* (in dose 500, 100, or 200 mg/kg body weight/day).	[184]
	*A. dracunculus*	Hydro-ethanol	Herb	nt	nt	In vivo (rats)	The extract (at dose 50, 100, or 200 mg/kg) decreased the levels of ALAT, ASPAT, alkaline phosphatase, and total bilirubin and increased total protein levels.	[40]
	*A. vulgaris*	Hydro-ethanol	Aerial part	nt	nt	In vivo (mice)	Prophylactic protective effect limiting inflammation, cellular edema, apoptotic cell count, and hyperemia of the hepatic parenchyma of hydro-ethanolic extract (at dose 600 mg/kg).	[209]
**Antispasmolytic activity**	*A. vulgaris*	Chloroform and methanol	Herb	Sesquiterpenoids	Yomogin and 1,2,3,4-diepoxy-11(13)-eudesmen-12,8-olide	In vivo (guinea pigs)	Antagonism toward H1 histamine receptors.	[138,142]
**Anthelmintic activity**	*A. absinthium*	Aqueous and an ethanolic	Aerial part	nt	nt	In vivo (sheep)	Extracts from *A. absinthium* (in dose 2 g/kg body weight) cause paralysis and/or death of *Haemonchus contortus* nematodes and reduce (80.49%) the number of the parasite’s eggs in the host’s feces.	[185]
		Essential oil	Aerial part	nt	nt	In vivo (mice)	Lethal effect on *Trichinella spiralis* larvae.	[86,186]
		Ethanolic	Herb	nt	nt	In vivo (rabbits)	Lethal effect of *A. absinthium* ethanolic extract on *Ascaris suum* eggs and *Trichostrongylus colubriformis* larvae.	[187]
		Ethanolic extract	Aerial part	nt	nt	In vivo (sheep), in vitro (parasite motility inhibition test)	Lethal effect on *H. contortus* tested in vivo; reduction in its mobility in vitro.	[188]
	*A. vulgaris*	Methanol	Herb	nt	nt	In vivo (rats)	Extract (at dose 300 mg/kg) inhibited activity against *T. spiralis* by 75.6% and 63.5% in the tongue, 53.4% and 37.7% in the diaphragm, 67.8% and 46.2% in the quadriceps, and 66.7% and 60.5% in the biceps–triceps muscles of rats.	[186]
**Antiprotozoal activity**	*A. absinthium*	Aqueous and ethanolic extracts	Aerial part	nt	nt	In vitro (mice)	Lethal effect of aqueous and ethanolic extracts from *A. absinthium* on *Plasmodium berghei* (in dose 74 mg/kg).	[25]
		Hydro-ethanolic	Herb	nt	nt	In vitro (chloroquine-resistant (K1) and chloroquine-sensitive (CY27) strains of *Plasmodium berghei)*	Lethal effect of the hydro-ethanolic extract *P. berghei.* IC_50_ *=* 0.46 μg/mL for the K1 strain and IC_50_ = 0.195 μg/mL for the CY27.	[26]
		nt	Herb powdered	nt	nt	In vivo (human)	Lethal effect of capsuled powdered herb of *A. absinthium* in dose 500 mg on *Entamoeba histolytica*.	[30]
		Essential oil	Aerial part	nt	nt	In vitro	Lethal activity against the promastigotes and amastigotes forms of the protozoa *Leishmania aethiopica* and *Leishmania donovani*. MIC for both microorganisms in the promastigote form was 0.1565 μL/mL.	[32]
		Ethanol	Aerial part	Flavonoids, sesquiterpenoid lactone	Artemetin, casticin, hydroxypelenolide	In vitro	Lethal activity in vitro against *Leishmania infantum* and *Trypanosoma cruzi*	[33,34]
		Essential oil	Aerial part	Sesquiterpenoids	(*E*)-Caryophyllene and 3,6-dihydrochamazulene	In vitro	Lethal effect of the essential oil on *T. cruzi* and on *Trichomonas vaginalis*. The compounds likely to be responsible for this activity are (*E*)-caryophyllene and 3,6-dihydrochamazulene.	[35]
		Aqueous and ethanolic	Aerial part	Sesquiterpenoids lactones	Artemisinin, dihydroartemisinin	In vitro	Inhibition (100%) of *Naegleria fowleri* growth by sesquiterpenoid lactones from *A. absinthium.*	[36]
		Aqueous	Aerial part	nt	nt	In vitro	Inhibition (88.9%) of *A. absinthium* aqueous extract against *Plasmodium falciparum*.	[37]
	*A. annua*	Methanol, ethanol, aqueous	Herb	Sesquiterpene lactone	Artemisinin	In vivo/In vitro	Lethal activity against *Artemisia* *castellani* of artemisinin and methanolic, ethanolic, and aqueous extracts from *A. annua* herb.	[27]
		n-Hexane, ethanol, and water	Leaves and seeds	nt	nt	In vitro	Compounds present in *A. annua* seed and leaf extracts have lethal activity against *L. donovani*.	[29]
	*A. d* *racunculus*	Hydro-ethanol	Herb	nt	nt	In vitro	The extract (at dose (100–1000 μg/mL) inhibited the development of the promastigote form of *Leishmania major*. The recorded MIC values of the extract after 24 h, 48 h and 72 h were: 962.03, 688.36 and 585.51 μg/mL.	[28]
**Immunostimulating activity**	*A. absinthium*	Ethanolic	Herb	nt	nt	In vivo (mice)	Induction of dendritic cell maturation by increasing the level of CD40 surface expression and by induction of cytokines. It was found that at 100 μg/mL extract the proliferation of T-lymphocytes was reduced by 78.2% relative to the control.	[189]
		nt	Herb	Polysaccharides	nt	In vivo (mice)	Induction of TH1 immune response and stimulation of nitric oxide production by macrophages.	[190]
**Immunosuppressive activity**	*A. annua*	Ethanol	Herb	nt	nt	In vitro/In vivo	Inhibition of lymphocyte proliferation and reduction in IgG, IgG1, and IgG2b antibody levels after the administration of *A. annua* whole-plant extract (at dose 0.25, 0.5, and 1. 0 mg).	[91]
		nt	Herb	Sesquiterpene lactone	Artemisinin	In vivo (mice)	Artemisinin obtained from *A. annua* inhibits late-type hypersensitivity response and has a suppressive effect on calmodulin responsible for activation of T lymphocytes.	[198]
	*A. d* *racunculus*	Aqueous	Herb	nt	nt	In vivo (mice)	The extract (at dose 100 mg/kg) reduced IL-17 (interleukin 17) and IFN-γ (interferon gamma) production and intensification of the phagocytosis process carried out by macrophages.	[149]
		Aqueous	Herb	nt	nt	In vivo (mice)	Lowering of IL-17 and IL-23 (interleukin-23) levels and reduction in the infiltration of leukocytes into brain cells.	[204]
		Hydro-ethanol	Leaves	nt	nt	In vivo (mice)	Increased neutrophil levels and decreased lymphocyte levels after intraperitoneal administration of the hydroethanolic extract from the leaves (at dose 200 mg/kg).	[205]
**Cytotoxic activity**	*A. absinthium*	Methanol	Leaves	nt	nt	In vitro	Inhibition of proliferation of breast cancer cells of MDA-MB-231 (50% at 20 g/mL) and MCF-7 lines (50%, at 25 g/mL).	[17]
		Essential oil	Aerial part	Sesquiterpenoids	*(E*)-Caryophyllene, germacrene D	In vitro	The essential oil, in particular (*E*)-caryophyllene and/or germacrene D, is toxic to tumor lines A548, NCI-H292, HCT116, MCF-7, and SK-MEL-5.	[18]
	*A. annua*	Ethyl acetate	Aerial part	Polyphenols	Caffeic acid, syringic aldehyde, dicaffeoylquinic acid isomer, quercetin 3-O-galactoside, dicaffeoylquinic acid isomer, mearnsetin 3-O-hexoside isomer, kaempferol 3-O-glucoside, quercetin 3-O-glucoside, ferulic acid, caffeoylferuloylquinic acid isomer, isorhamnetin 3-O-glucoside, diosmetin 7-O-glucoside, luteolin 7-O-glucoside, diferuloylquinic acid, quercetin, dicaffeoylferuloylquinic acid isomer, 3-O-methylquercetagetin, luteolin, 8-methoxykaempferol, 3,5-dimethoxyquercetagetin, caffeoyldiferuloyl quinic acid, kaempferol, 3,5-dihydroxy-6,7,4′-trimethoxyflavone, and 3,5-dihydroxy-6,7,3′,4′-tetramethoxyflavone	In vitro	Polyphenols present in *A. annua* inhibit adhesion of cancer cells to endothelial cells and inhibit epithelial–mesenchymal transition.	[123]
		nt	Herb	Sesquiterpenoid lactone	Artemisinin	In vivo	Regression of prostate cancer in a patient treated (at dose 5 mg/day) with capsules containing a concentrate with *A. annua* and bicalutamide.	[199]
		Methanol	Leaves	nt	nt	In vitro	Methanolic extract from *A. annua* leaves collected in Egypt showed significant cytotoxic activity against MCF-7 human breast adenocarcinoma cell line, human lung cancer cell line, and Chinese hamster ovary (CHO) cell line.	[201]
	*A. vulgaris*	Methanol	Aerial part	nt	nt	In vitro	Inhibition of tumor cell growth in cancer cell lines: MCF-7 (IC_50_ = 190 ng/mL), HeLa (IC_50_ = 284 ng/mL), A7R5 (IC_50_ = 382 ng/mL), 293T (IC_50_ = 317 ng/mL), and SW-480 (IC_50_ = 778 ng/mL).	[210,211,212]
**Analgesic activity**	*A. absinthium*	Methanolic	Herb	nt	nt	In vivo (mice)	Reduction in temperature-induced pain in mice at doses of 300 mg/kg, 500 mg/kg or 1000 mg/kg.	[191]
		Essential oil/Aqueous	Aerial part	nt	nt	In vivo (mice)	Reduction in episodes in the writhing test and delay in pain response in the hot plate test in mice after the administration of *A. absinthium* essential oil (at doses of 2, 4, or 8 mg/kg) or aqueous extract (50, 100, or 200 mg/kg).	[122]
	*A. annua*	Essential oil	Herb	Monoterpenoids	Camphor, 1,8-cineol, and α-pinene	In vivo (mice)	Administration of essential oil (at dose 400 mg/kg) from *A. annua* herb, camphor, 1,8-cineol, and α-pinene in mice reduces (57%) writhing episodes caused by acetic acid.	[93]
	*A. vulgaris*	Hydro-ethanol	Aerial part	Flavonoids, phenolic acids	Rutoside, hydroxybenzoic acid derivatives, and caffeic acid and its derivatives.	In vivo (mice)	Mild peripheral antinociceptive effect of extract (at dose 100 and 250 mg/kg).	[142]
**Inhibiting the activity of carbonic anhydrase I and II**	*A. dracunculus*	Dichloromethane	Herb	Phenylpropanoid derivatives, sterols, coumarin	*trans*-Anethole, stigmasterol, herniarin, (2E,4E)-N-isobutylundeca-2,4-diene-8,10-diynamide, (2E,4E)-1-(piperidin-1-yl)undeca-2,4-diene-8,10-diyn-1-one and 1-(4’-methoxyphenyl)-1,2,3-trihydroxypropane	In vitro	Compounds present in herbal extracts reduce the activity of carbonic anhydrase I (hCA I) and II (hCA II) (IC_50_ = 0.02 μg/mL for hCA I, and IC_50_ = 0.31 μg/mL for hCA II).	[216]
**Neuroprotective activity**	*A. absinthium*	Methanol	Aerial part	nt	nt	In vivo (rats)	Methanolic extract (at dose 100 and 200 mg/kg) from *A. absinthium*, because of its antioxidant potential, reduces brain damage, inhibits lipid peroxidation, and restores the activity of enzymes involved in reducing oxidative stress.	[14]
		Aqueous	Herb	nt	nt	In vivo (rats)	Protective effect of *A. absinthium* aqueous extract (at dose 200 mg/L) on glial cells and the dopaminergic system when exposed to lead.	[15]
			Herb	Sesquiterpenoid dimer	Caruifolin D	In vitro (BV2 microglial cells)	Caruifolin D in *Absinthii herba* inhibits the production of proinflammatory microglia mediators and reactive oxygen species and also inhibits protein C kinase and stress-activated kinases.	[130]
**Antidepressant activity**	*A. absinthium*	Methanol	Aerial part	nt	nt	In vivo (mice)	Shortening of the period of mouse immobility in the forced swim test (at dose 1000 mg/kg) and in the tail suspension test (at dose 500 mg/kg).	[16]
	*A. dracunculus*	Ethanol	Herb	nt	nt	In vivo (mice)	Increased resistance to stressful situations and reduction in stress-related levels of inflammatory cytokines.	[206]
		Ethanol	Herb	Phenolic acids, flavonoids	Chlorogenic acid, caffeic acid or luteolin and quercetin	In vivo (mice)	Phenolic compounds and flavonoids contained in the *A. dracunculus* herb extract (at dose dose of 200 mg/kg) reduce the immobility response time in mice in the writhing test and in the forced swim test.	[114]
		Ethanol	Herb	Coumarins	Herniarin, skimmin c	In vitro	Mild inhibition of hMAO-A (human monoamine oxidase A) and hMAO-B (human monoamine oxidase B) by extracts of *A. dracunculus* and compounds. Herniarin and skimmin c showed the inhibitory effects against *h*MAO A (IC_50_ = 51.76 and 73.47 μM, respectively) and *h*MAO B (IC_50_ = 0.84 and 1.63 mM, respectively).	[112]
**Procognitive activity**	*A. absinthium*	Ethanol	Aerial part	nt	nt	In vitro (human cortical brain cells)	Extract in concentration 29 mg/mL had affinity for human muscarinic (99.8%) and nicotinic receptors (99.8%) responsible for cognitive functions.	[38]
**Neurotrophic activity**	*A. absinthium*	Methanol, ethanol and aqueous	Aerial part	nt	nt	In vitro (PC12D cells (cell line of rat pheochromocytoma tumor)	Methanolic, ethanolic, and aqueous extracts from *A. absinthium* induce the nerve growth factor, which stimulates development of neurites.	[217]
**Nephroprotective activity**	*A. annua*	Essential oil	Aerial part	nt	nt	In vivo (rats)	Administration of *A. annua* essential oil to rats exposed to carbon tetrachloride prevents kidney damage.	[93]
**Stabilizing cell membrane activity**	*A. absinthium*	Hydroalcoholic	Aerial part	nt	nt	In vitro	Hydroalcoholic extract from *A. absinthium* prevents hemolysis of erythrocytes.	[218]
**Auxiliary action in obesity treatment**	*A. annua*	Essential oil	Aerial part	nt	nt	In vitro	Reduction in fat droplet accumulation and inhibition of PPARγ (peroxisome proliferator- activated receptor gamma), C/EBPα (CCAAT/enhancer-binding protein), SREBP-1c (Sterol regulatory element-binding protein 1), FAS, and ACC (Acetyl-CoA carboxylase) protein expression under the influence of *A. annua* essential oil.	[202]
		Hydro-ethanol	Leaves	nt	nt	In vivo (mice)	Reduction in insulin resistance, liver steatosis, and fibrosis. Lowering the levels of SREBP-1c, ChREBP (carbohydrate-responsive element-binding protein), and COX-2 (cyclooxygenase-2). Inhibition of TGF-β1 and connective tissue growth factor.	[203]
**Hypoglycemic activity**	*A. dracunculus*	Ethanol	Herb	nt	nt	In vivo	Encapsulated ethanolic extract of *A. dracunculus* (at dose 1000 mg for 90 days) decrease in glycated hemoglobin (5.8% in the control group, 5.6% in the test group), area under the curve for insulin (56.136 to 27.426 pmol/L in the control group, 44.472 to 23.370 pmol/L in the test group), total insulin secretion (0.45 to 0.23 in the control group, 0.35 to 0.18 in the test group), and systolic blood pressure (120 mm Hg in the control group, 113 mmHg in the test group), and increase in HDL-C.	[207]
**Hypolipemic activity**	*A. vulgaris*	Aqueous	Root	nt	nt	In vivo (rat)	Normalized serum lipid profile, a significant increase in paraoxonase-1 activity, and decrease in serum malondialdehyde, nitric oxide, and tumor necrosis factor-α levels and in hydroxymethylglutaryl-CoA reductase activity. Lowering total cholesterol, triglycerides, LDL (low-density lipoprotein), and VLDL (very low density lipoprotein), and increasing HDL (high density lipoprotein) and atherogenicity indicator (aqueous extract of *A. vulgaris* roots).	[213,214]
**Antihypertensive activity**	*A. vulgaris*	Aqueous and chloroform	Aerial part	nt	nt	In vivo (rats)	A 10% solution of the aqueous extract inhibiting the hypertensive effect of noradrenaline.	[215]
**Bronchodilatory activity**	*A. vulgaris*	Methanol	Aerial part	Alkaloids, coumarins, flavonoids, saponins, sterols, tannins, and terpenoids	nt	In vivo (rabbit jejunum and guinea pig trachea)	Anticholinergic and Ca^2+^ antagonist mechanisms. Histamine H1 antagonism in the ileum and trachea.	[138,208]
**Normalizing the profile of thyroid hormones**	*A. dracunculus*	Aqueous	Herb	nt	nt	In vivo (rats)	Extract (at dose 300 mg/kg) caused increase in thyroxine and triiodothyronine levels, decrease in thyrotropin levels, increase in total antioxidant capacity, increase in glutathione, and decrease in malondialdehyde levels.	[22]
**Estrogenic activity**	*A. vulgaris*	Ethyl acetate	Aerial part	Flavonoids	Eriodictyol and apigenin	In vivo (rats)	Antagonism toward the estrogen receptor and activation of gene transcription. Induction of gene transcription by eriodictyol and apigenin. Anti-implantation activity and estrogenic activity on female Wistar rats.	[23,24]
**Insect repellent activity**	*A. abrotanum*	Toluene extract	Herb	Monoterpenoids, coumarins, phenolic acids	Camphor, coumarin and thujyl alcohol, chlorogenic acid and caffeic acid	In vivo	Toluene extract from the herb *A. abrotanum* and the individual components of the extract showed an insect repellent effect against *Ixodes ricinus* and *Aedes aegypti*. After 4 and 8 h from the time of applying the ethanolic suspension of the toluene extract from the herb *A. abrotanum*, the recorded repellency rates were, respectively, 69.1% and 56.8% against *Ixodes ricinus*, and 100% and 86.7% against *Aedes aegypti*.	[116]
	*A. dracunculus*	Essential oil	Herb	nt	nt	In vitro	Inhibition of *Calliphora vomitoria* egg laying on fresh beef, on which the essential oil of *A. dracunculus* herb (at dose 0.05 μL/cm^2^) was applied.	[96]
		Essential oil	Herb	nt	nt	In vitro	Larvacidal effect against *Anopheles stephensi* under the influence of nanoemulsion of *A. dracunculus* essential oil (consisting of 0.35% tarragon oil, 10% of Tween 20 and deionized water).	[102]
**Anti-animal parasites activity**	*A. abrotanum*						Reduction in the number of eggs of *Hymenolepis nana* (dwarf tapeworm), *Syphacia obvelata*, and *Aspiculuris tetraptera* (rodent pinworms) in the feces of mice after administration of ethanolic extract from *A. abrotanum* leaves.	[192]
	*A. annua*	Water, 0.1% sodium bicarbonate solution, dichloromethane, and methanol	Leaves	Sesquiterpenoid lactones	Artemisinin	In vivo	Extracts from *A. annua* leaves inhibit the growth of larvae and the hatching of eggs of *Haemnochus contortus* (parasite of sheep and goats).	[118]
**Antiplasmodial activity**	*A. abrotanum*	Ethanol/water (1/1)	Leaves	nt	nt	In vitro/Hemolysis assay	Notable antiprotozoal activity against *P. falciparum* under the influence of *A. abrotanum*-AgNPs in concentration ranging from 0.6 to 7.5 µg/mL. The inhibition dependent on concentration was 50%, 90%, and 99%.	[219]
**Antimalarial activity**	*A. annua*	Methanol	Herb	nt	nt	In vivo	Improvement in malaria symptoms after treating patients with infusion of *A. annua* herb. Inactivation of protozoan calcium pump.	[193]
		Hydro-ethanol and aqueous	Leaves	nt	nt	In vivo	Lethal activity of hydroethanolic and aqueous extracts from *A. annua* leaves (at dose 20 mg/kg) against *P. falciparum* and *P. berghei*.	[194]
		nt	Herb	Sesquiterpenoid lactones	Artemisinin	In vitro	Interference of artemisinin with protein metabolism and mitochondrial activity of *Plasmodium* spp. protozoa.	[195]
		nt	Leaves	Sesquiterpenoid lactones	Artemisinin	In vitro	Synergism of action of artemisinin and other compounds present in *A. annua* leaves against *P. falciparum*.	[131]
	*A. vulgaris*	Ethanol	Leaves	nt	nt	In vitro	Activity against *Plasmodium yoelii* and *P. berghei.* The extract at doses of 500, 750, and 1000 mg/kg significantly inhibited parasitemia by 79.3%, 79.6%, and 87.3%, respectively.	[220,221]

* nt—not tested.

**Table 6:** Possible applications of *Artemisia* species in cosmetology as recommended by the CosIng database [231].

Species	INCI Name	Description	Functions
*A. abrotanum*	*Artemisia abrotanum* extract	Extract of the whole plant of the Southernwood, *A. abrotanum*	Skin protecting
	*Artemisia abrotanum* leaf/stem extract	Extract of the flowers, leaves, and stems of the Southernwood, *A. abrotanum*	Moisturizing Skin conditioning
*A. absinthium*	*Artemisia absinthium* extract	Extract of the whole herb of the Wormwort, *A. absinthium*	Skin conditioning
	*Artemisia absinthium* herb extract	Extract obtained from the flowering herb of the Wormwort, *A. absinthium*	Perfuming
	*Artemisia absinthium* herb oil	“Wormwood Oil”, essential oil obtained from the flowering herb of the Wormwort, *A. absinthium.* It contains thujyl alcohol, thujyl acetate, thujone, phellandrene, cadinene, and a blue oil	Perfuming
	*Artemisia absinthium* oil	Volatile oil obtained from the whole plant of the Wormwort, *A. absinthium*	Antimicrobial
	*Artemisia absinthium*/*Chamaecyparis obtusa* wood extract	Extract of the whole plant, *A. absinthium*, and the wood of *C. obtusa*	Antimicrobial Hair conditioning Skin conditioning—emollient
*A. annua*	*Artemisia annua* (leaf/stem)/*Ficus carica* fruit/*Ginkgo biloba* leaf extract	Extract of the leaves and stems of *A. annua*, the fruit of *F. carica*, and the leaves of *G. biloba*	Skin conditioning
	*A. annua* callus extract	Extract of the callus of *A. annua* grown in culture	Antimicrobial Antioxidant Hair conditioning Skin conditioning Skin protecting
	*Artemisia annua* extract	Extract of the whole herb, *A. annua*	Fragrance
	*Artemisia annua* flower/leaf/stem extract	Extract of the flowers, leaves, and stems of *A. annua*	Skin conditioning—miscellaneous
	*Artemisia annua* herb oil	Essential oil obtained from the whole herbs of the plant *A. annua*	Perfuming
	*Artemisia annua* leaf extract	Extract obtained from the leaves of the plant *A. annua*	Antiseborrheic Antimicrobial Perfuming Skin conditioning
	*Artemisia annua* leaf/stem extract	Extract of the leaves and stems of *A. annua*	Skin conditioning
	*Artemisia annua* meristem cell extract	Extract of the cultured meristem cells of *A. annua*	Antioxidant
	*Artemisia annua* oil	Volatile oil obtained from the whole plant, *A. annua*	Antioxidant Humectant Skin conditioning Skin conditioning—emollient
	*Artemisia annua* seed extract	Extract of the seeds of *A. annua*	Antioxidant
	*Artemisia annua*/*Citrus junos* fruit/*Pinus densiflora* leaf extract	Extract of the whole plant *A. annua*, the fruit of *C. junos*, and the leaves of *P. densiflora*	Skin protecting
*A. dracunculus*	*Artemisia dracunculus* flower	Flower of *A. dracunculus*	Skin conditioning
	*Artemisia dracunculus* herb extract	Extract obtained from the whole herb of the Tarragon, *A. dracunculus*	Perfuming
	*Artemisia dracunculus* leaf/stem extract	Extract of the leaves and stems of the Tarragon, *A. dracunculus*	Fragrance
	*Artemisia dracunculus* oil	Essential oil obtained from the whole herbs of the Tarragon, *A. dracunculus*	Perfuming Skin conditioning
	*Artemisia dracunculus* root extract	Extract of the roots of the Tarragon, *A. dracunculus*	Skin conditioning
	*Artemisia dracunculus* seed/*Anthemis nobilis* seed/*Hypericum androsaemum* seed extract	Extract of the seeds of the Tarragon, *A. dracunculus*, *A. nobilis*, and *H. androsaemum*	Skin conditioning
*A. vulgaris*	*Artemisia vulgaris* extract	Extract of the whole plant of the Common Mugwort, *A. vulgaris*	Skin conditioning
	*Artemisia vulgaris* herb extract	Extract obtained from the whole herb of the Common Mugwort, *A. vulgaris*	Perfuming
	*Artemisia vulgaris* leaf extract	Extract of the leaves of *A. vulgaris*	Antioxidant Skin conditioning—emollient Skin protecting
	*Artemisia vulgaris* oil	Volatile oil obtained from the whole herb of the Common Mugwort, *A. vulgaris*	Perfuming Skin conditioning
